# Factors and modification techniques enhancing starch gel structure and their applications in foods:A review

**DOI:** 10.1016/j.fochx.2024.102045

**Published:** 2024-11-26

**Authors:** Yongqiang Gong, Shuzhi Xiao, Zihan Yao, Hongjie Deng, Xuan Chen, Tao Yang

**Affiliations:** a College of Food Science and Engineering, Central South University of Forestry and Technology, Changsha 410004, China; b School of Architecture and Art, Central South University, Changsha 410004, China

**Keywords:** Starch gels, Starch composition, Retrogradation conditions, Food ingredients, Modifications

## Abstract

The formation of starch gel structure results from the gelatinization and retrogradation of starch in aqueous solutions, which plays a crucial role in determining the quality and functional properties of starchy foods. The gelation ability of many native starches is limited and their structure is weak, which restricts their application. Therefore, how to enhance the gel structure of starch is of great significance to food science and industry. In this paper, the mechanism of starch gel formation was reviewed, and the research progress of starch composition, retrogradation conditions, food composition and modification methods were reviewed. Meanwhile, the applications of enhanced starch gel structures in food quality, nutrition, packaging, and 3D printing were discussed. This review provides valuable references for researchers and producers to develop high-quality and nutritious starch-based foods and further expand the applications of starches.

## Highlights

• Starch gels are formed by gelatinization and retrogradation of starch.
• Starch gel structure can be strengthened by the selection of starch sources, rerogradation conditions and food ingredients.
• The related modification techniques for strengthening starch gel structure were summarized.
• Advances in the food application of starch gel structure enhancement were provided.

## 1 Introduction

Starch serves as the primary energy source for human metabolism and is an essential ingredient in starch-based foods (Nagasaki et al., 2021). Starch particles are composed of amylose and amylopectin, including crystalline and amorphous regions. Amylose is a linear chain of glucopyranose units linked by α-1,4-glycosidic bonds, while amylopectin has a branching structure with linear branches linked by α-1,4-glycosidic bonds and periodic α-1,6-glycosidic linkages (Chakraborty et al., 2022). The starch suspension in water is heated to a high concentration to form a dense paste through the gelatinization process. Starch gels are complex networks formed by the recombination of gelatinized starch molecules during cooling (Hu et al., 2020). Due to their exceptional ability to form well-structured gel networks, produce uniform pastes, thicken, and provide energy, starch gels are the most commonly used food hydrocolloid. In the development of numerous food formulations, starch gels play a crucial role in determining physicochemical and functional properties. A starch gel structure with sufficient strength enhances the mechanical properties of starch-based foods, meeting processing requirements (Hirao et al., 2021; Ulbrich et al., 2023). In recent years, researchers have paid more and more attention to the potential of starch gel structures in regulating the structure and performance of foods.

Rheological properties, texture properties, and microstructure, among others, are important indicators used to evaluate starch gel structure (Li, Hu, et al., 2020). The enhancement of gel structure is specifically manifested in increased storage modulus (G′) and loss modulus (G″) in rheology, as well as a smaller tan α (Adamczyk et al., 2022). For texture properties of starch gels, gel strength and hardness increase (Liu et al., 2021). The gel network becomes more orderly, and the gaps are more compact in the microstructure (Pan et al., 2024). Additionally, starch granules swell and gelatinize with sufficient moisture and heating, allowing starch molecules to interconnect, which forms the basis of the gel structure (Jia et al., 2023; Li, 2022). The enhancement of starch gel structure results from increased cross-linking between starch molecules, which is a desirable trait for many starch-based foods. It overcomes the shortcomings of starch gel processing performance and improves stability during processing, which enhances product quality (Fan et al., 2022). For instance, vermicelli require moderate retrogradation properties of the gel, good freeze-thaw stability, low swelling power, and breakdown viscosity, strong gel strength, and high light transmittance (Chen et al., 2022). Pastry cream or custard requires certain stretchability and hardness, which requires a gel with high viscoelasticity and shape retention (Hirao et al., 2021).

In the past, researchers have focused on reducing hydrogen bonding interactions between starch molecules, which is accompanied by a weakening of the starch gel structure and a delay in food quality deterioration caused by starch retrogradation. Wang et al. (2015) and Chang et al. (2021) conducted comprehensive reviews on retrograded starch, indicating that starch retrogradation can reduce the shelf life and consumer acceptability of starch-based foods, leading to considerable waste. In fact, some starch-based foods require strong interactions between starch molecules to enhance the network structure of starch gels, which improves the stability of starch-based foods, improving digestive ability, and enhancing mechanical processing properties (Mohamed, 2023). However, the ability of some natural starches to form a gel structure is limited. Although many researchers have explored the gel network structure formed after starch gelatinization, no review has specifically summarized how to enhance the starch gel structure and its applications in foods. In this paper, the mechanism of starch gel formation was reviewed, and the factors affecting the structure of starch gel, such as starch composition, retrogradation conditions and food composition, were discussed. In addition, how to enhance the structure of starch gel by physical, chemical and enzymatic modification techniques was discussed, and its application in food science was reviewed.

## 2 Formation mechanism of starch gels

**Fig. 1:**
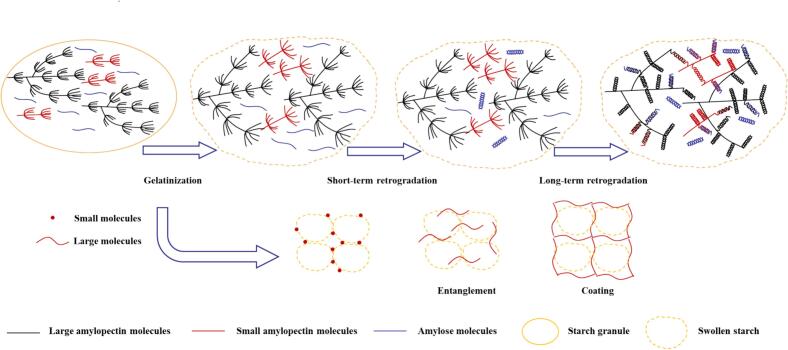
The formation mechanism of starch gels.

The conversion of native starch granules into starch gels involves two distinct processes: gelatinization and retrogradation. As illustrated in Fig. 1, gelatinized starch undergoes a cooling process, which promotes retrogradation and leads to the formation of a gel state. At room temperature, adding cold water to starch forms a water-insoluble suspension with limited and reversible swelling of starch particles (Chakraborty et al., 2022). When heat is applied, water molecules enter the amorphous regions of the starch, leading to the gradual leaching of amylose within these domains (Ji et al., 2022). The relatively stable structure of the crystalline regions is disrupted by the hydration and swelling of starch when heated beyond the gelatinization temperature. The hydrogen bonds in both the crystalline and amorphous regions of starch molecules are broken, allowing new hydrogen bonds to form between water and starch molecules (Xu, Blennow, et al., 2020). Once the starch particles have absorbed sufficient water, they disintegrate and dissolve, forming a disordered gel network. In this process, amylose acts as the dispersing medium, while amylopectin serves as the dispersed phase, marking the completion of gelatinization (Nagasaki et al., 2021). Starch gelatinization involves the infiltration of water molecules into the starch crystallites, creating hydrogen bonding interactions with starch molecules. This disrupts the initial association state and induces a rearrangement from a relatively ordered to a disordered conformation (Wang et al., 2016). Gelatinized starch with higher amylopectin content demonstrates increased instability compared to amylose due to greater steric hindrance between starch chains (Li et al., 2021). Consequently, the formation of hydrogen bonds is impeded, prolonging the crystallization process (Chang et al., 2021). During the cooling of gelatinized starch, amylose and amylopectin gradually reassociate to form ordered structures, ultimately resulting in gel formation (Wang et al., 2022). In contrast to gelatinization, starch retrogradation is a process wherein starch molecules transition from a disordered state to an ordered state during the cooling phase of the starch paste (Huang et al., 2021). Retrogradation can be categorized into short-term and long-term retrogradation. Short-term retrogradation primarily involves the formation of a network structure through directional movement and aggregation between amylose molecules, which predominantly occurs during the initial cooling phase of the starch paste (Chang et al., 2021). Long-term retrogradation occurs several days or even weeks after the starch paste has cooled, primarily due to the reassociation of amylopectin molecules, further enhancing the structure of starch gels (Li, Yang and Li, 2022). Starch gels form through the synergistic effect of amylose and amylopectin retrogradation. Amylose reassociation produces crystals that act as the crystalline nucleus, while amylopectin reassociation forms a crystalline region that grows around the nucleus (Ulbrich et al., 2023). Small amylopectin molecules, released from starch particles during gelatinization, enhance the gel network structure, whereas large amylopectin molecules hinder the gel network structure by limiting the release of long amylose chains from starch particles (Ulbrich et al., 2023).

Amylopectin molecules typically have struggle forming double helices through intramolecular interactions due to the high steric hindrance of the α-1,6-glycosidic linkage (Li et al., 2021). However, evidence suggests that longer outer chains and smaller amylopectin molecules can generate both intermolecular and intramolecular interactions during long-term retrogradation, owing to their high flexibility (Bertoft et al., 2016; Martinez et al., 2018). Furthermore, the internal chains of amylopectin can participate in forming intermolecular interactions during retrogradation, contributing to the formation of starch gels with a more compact microstructure. While amylose typically forms long-range double helices through both intramolecular and intermolecular interactions, it has also been shown to interact with amylopectin molecules, forming short intermolecular double helices during retrogradation (Bertoft et al., 2016; Li, Yu and Gilbert, 2022).

In reality, starch gels in foods rarely exist as pure starch. Even at low concentrations, proteins and lipids present in native starch significantly impact the formation of starch gel networks (Tao et al., 2024). Additionally, gel performance is substantially influenced by interactions between other components and starch through hydrogen bonding, electrostatic, and hydrophobic interactions (Li, 2022). Macromolecular substances, such as polysaccharides and proteins, can contribute to starch gel formation through entanglement or coating, as illustrated in Fig. 1. Moreover, the formation of starch gels is also affected by retrogradation conditions and modifications.

## 3 Factors enhancing starch gel structure

### 3.1 Starch composition

The composition of starch significantly affects the formation of starch gel structures (Li et al., 2021), as summarized in Table 1. Extensive research shows that amylose plays a dominant role in the early stages of gel formation by promoting crystallization. During retrogradation, amylose promotes crystallization, which leads to a compact and ordered network that enhances gel strength (Tian et al., 2023; Ulbrich et al., 2023). The linear molecular characteristics of amylose facilitate faster migration rates and require less space for rearrangement and relocation. In contrast, the highly branched structure of amylopectin complicates reorganization, resulting in disordered starch chains after gelatinization (Li, Yu and Gilbert, 2022; Tian et al., 2023; Wang, Liu, & Ai, 2022). In addition, longer amylose molecules with extended chain lengths and smaller molecular sizes, combined with a lower amylopectin unit-chain ratio, were found to enhance the hardness, elasticity, and tensile strength of rice noodles (Zhang et al., 2022). This improvement is due to the ability of longer amylose molecules to traverse multiple crystalline lamellae of amylopectin and interact with it. Furthermore, the smaller molecular size of amylose promotes the formation of a firmer gel by facilitating leaching and rearrangement during gelatinization (Zhang et al., 2022). However, amylose also exhibit anti-gelatinization properties. Amylose in corn starch granules showed a pronounced inhibitory effect on granule expansion and exhibited strong anti-gelatinization properties (Zhao et al., 2022) Additionally, amylose promotes the formation of starch-lipid complexes during gelatinization, preserving significant long-range and short-range ordered structures during heating. In a comparative study of the viscoelasticity of mung bean and proso millet starches, it was found that amylopectin long chains more easily cross-linked to form a stable network structure, which was a key factor affecting the viscoelasticity of starch gels (Qiao et al., 2024). This finding is consistent with Zhou et al. (2021). The unit chains were found to be more ordered than the smaller amylopectin molecules and were more likely to interact with both themselves and amylose (Zhang et al., 2022). Li et al. (2019) reported that amylopectin size significantly impacted the quality of rice starch gels and amylopectin with smaller amylopectin molecules caused greater viscoelasticity and a stronger network. This effect was due to reduced resistance of smaller amylopectin molecules to amylose precipitation from starch particles during heating. Taken together, the ability to predict the gel structure of starch can be improved by taking into account the compositional properties and complex structure of starch. This understanding contributes to the strategic selection of appropriate starch substrates to formulate starch-based foods and allows precise manipulation of amylose and amylopectin components, thereby improving the overall quality of the final product.

**Table 1:** Factors of starch composition enhancing starch gel structure.

Factors	Starch source	Conditions	Results	References
Composition	High amylose corn starch, lutinous rice, Japonica rice, Indica rice	High amylose content.	Gel strength enhanced.	Tian et al., 2023; Ulbrich et al., 2023
	Cake batter	viscoelasticity showed a positive correlation with the length of amylose intermediate and long chains as well as amylopectin long chains.	Gel strength and elasticity enhanced.	Yang, Pan, et al., 2022
	Rice noodle	Length chain and smaller molecular size of amylose, amylopectin with a lower amylopectin unit-chain ratio.	Hardness, elasticity, and tensile strength of rice noodle increased.	Zhang et al., 2022
	Mung bean starch, proso millet starch, waxy maize starch, amylose-extender waxy maize starch	Amylopectin with a higher proportion of longer external chains.	Exhibited higher viscoelastic properties, formed a stable network structure.	Qiao et al., 2024; Zhou et al., 2021
	Rice starch	Smaller amylopectin.	Greater viscoelasticity and stronger network.	Li, Lei, et al., 2019
	Indica rice starch	GCPs and SGAPs were selectively removed.	Leading to an increase in the leaching of linear starch, a decrease in both the G′ and G″, and a weakening of the gel structure.	Luo & Wang, 2022
	Rice starch		GCPs exert a more significant influence on the formation of starch gels than surface proteins.	Zhan et al., 2020
	Buckwheat starch		The starch aggregates and the gel strength significantly decreased	Du et al., 2024
	Rice starch	The elimination of lipids via sodium dodecyl sulfate treatment diminishes the concentration of linear starch-lipid complexes.	Culminating in an increase in the swelling power of rice starch.	Hu et al., 2017
	Cross-linked glutinous corn starch.	Removed the surface lipids	Viscoelastic properties (G′ and G″) enhanced.	Xu, Liu, et al., 2024

During the extraction of pure starch, most proteins are removed, leaving behind residual proteins known as starch granule-associated proteins (SGAPs), which include surface proteins and channel proteins located within starch granules (Chen, McClements, et al., 2024). Recent studies have shown that despite their minimal presence, SGAPs contribute to starch gel formation (Du et al., 2024). Luo and Wang (2022) demonstrated that SGAPs occupied the channels within indica rice starch granules and the intergranular spaces, which formed a polymeric skeleton and constructed a starch gel network. The selective removal of granular channel proteins (GCPs) and SGAPs resulted in the increase of amylose leaching, the decrease of G′ and G′, and the decrease of gel structure. Interestingly, the effect of GCPs in rice starch granules on starch gel formation was more significant than that of surface proteins (Zhan et al., 2020). Similar findings were observed in another study, where the removal of SGAP from buckwheat starch significantly reduced starch aggregates and gel strength (Du et al., 2024). A different perspective was provided by Ma, Zhu, et al. (2022), who studied oat, quinoa, amaranth, and rice starches, showing that removing SGAP contributed to increased amylose leaching and accelerated the recrystallization and rearrangement of starch molecules, resulting in a stronger gel network. Moreover, starch-lipid complexes, also known as V-type crystalline starch, are typically present in native starch or synthesized during starch gelatinization. Higher concentrations of these complexes are detrimental to gel formation, as they hinder the hydration of linear starch molecules and can create a hydrophobic layer on the granule surface, obstructing interactions between starch granules and water. In a relevant study, Hu et al. (2017) concluded that removing lipids using sodium dodecyl sulfate treatment reduces the concentration of linear starch-lipid complexes, resulting in increased swelling power of rice starch. Similarly, Xu, Wang, et al. (2024) found that removing surface lipids significantly improved the viscoelastic properties (G′ and G″) of cross-linked glutinous corn starch.

### 3.2 Retrogradation conditions

#### 3.2.1 Gelatinization temperature

Some starch-based foods can achieve their optimal characteristics without complete starch gelatinization by carefully controlling the temperature during processing (Chakraborty et al., 2022). During starch gelatinization, the degree of swelling progressively increases with rising temperature. Once the temperature exceeds the gelatinization point, substantial swelling occurs. A comprehensive study investigated the effects of increasing heating temperatures on the physicochemical properties of starch (Cheng, Chen, et al., 2023), as detailed in Table 2. The study revealed that even at gelatinization temperatures of 65 °C and 70 °C, the partial crystalline struture of starch particle was retained, with most starch molecular chains remaining confined within these particles. This confinement impeded the formation of starch gel networks, resulting in a low G′ value. As the temperature increased further, amylose gradually precipitated out, with the highest G′ value notably observed at 80 °C. However, as the gelatinization temperature continued to rise beyond the optimal point, excessive heat reduced interactions among effective starch chains and caused the shortening of amylopectin molecules. This instability weakened the structural strength and compromised the integrity of the gel network. Similar findings were reported in a study on potato starch, where excessively high temperatures led to a compromised gel structure (Torres et al., 2018). Although temperatures above the gelatinization point generally promote gel formation, overheating can break starch chains, resulting in a weakened gel network.

**Table 2:** Enhancement of starch gel structure by controlling retrogradation conditions.

Factors	Starch source	Results	References
Gelatinization temperature	Corn starch	Corn starch showed low G' at gelatinization temperatures of 65 °C, and 70 °C, exhibited the highest G' at 80 °C, as the gelatinization temperature continued to rise, G' decreased.	Cheng, Liang, et al., 2023
Storage temperature	Potato starch	(1) Hardness, viscosity, and chewiness increased with decreasing aging temperature (Aging temperature exceeded 9 °C), formed a uniform network structure at 3 °C and 0 °C. (2) Hardness and chewiness of the starch gels decreased when the aging temperature dropped below −9 °C.	Jiang et al., 2020
	Cassava starch	(1) An increase in both G' and G" as the duration of freezing time increased prior to reaching 200 min. (2) After 200 min of freezing time, water-holding capacity and viscoelasticity decreased, and the submicrostructure became looser.	Xu, Zhu, & Yi, 2023
	Oat roll starch	Rapid freezing at −40 °C and − 80 °C inhibited ice crystal enlargement and structural rupture, thus maintaining internal structural integrity.	Gong et al., 2020
pH	HMT cassava starch	(1) HMT cassava starch gel pretreated by acid soaking (pH 3) was white-turbid, non-sticky and spoonable (yoghurt-like). (2) HMT cassava starch gel pretreated by alkali soaking (pH 11) was brown-turbid, nonsticky, stable and spoonable (pudding-like).	Chatpapamon et al., 2019
	Pregelatinized and granular cold water swelling corn starches	(1) The texture parameters, turbidity and freeze-thaw stability of the samples decreased, while the solubility increased at pH 3 and 5. (2) The rheological and mechanical properties, freeze-thaw stability and turbidity of the starch paste increased at pH 9 and 11.	Hedayati et al., 2016
	Waxy potato starch	Waxy potato starch showed a high G' and opaque gel appearance under acidic conditions (pH 4–6), but showed the opposite gel properties under acidic conditions (pH 8–10).	Fang et al., 2020

#### 3.2.2 Storage temperature

There is a clear correlation between storage temperature and the properties of starch gel in its non-frozen state (Table 2). The degree and rate of retrogradation vary depending on temperature. At 4 °C, the formation of a crystalline nucleus is increased, while crystal growth is decreased. Conversely, at room temperature (25 °C), crystalline nucleus formation is hindered, but crystal growth is facilitated (Chang et al., 2021). The retrogradation rate of bread starch in the non-freeze-thaw state was found to accelerate as storage temperature decreased, resulting in reduced water-holding capacity and an increased recrystallization rate after freeze-thaw cycles (Chang et al., 2021). An investigation into the gel properties of potato starch at an 8 % concentration showed that as the aging temperature dropped below 9 °C, the hardness, viscosity, and chewiness of the gels increased (Jiang et al., 2020). Microstructural analysis showed that gels formed at 3 °C and 0 °C developed a firm, fine, and uniform network structure, was likely due to the optimal aging temperature for starch being between 10 °C and − 3 °C. However, temperatures below −9 °C led to increased hardness and chewiness, attributed to ice crystal formation. These ice crystals disrupted the network structure of potato starch gels, leading to a sponge-like or semi-sponge structure. Xu, Zhu, and Yi (2023) further investigated the effect of freezing on the structure of starch gels, focusing on cassava starch gels frozen at −20 °C. Before freezing, an increase in both G′ and G″ was observed as freezing time increased. This was attributed to an increase in short-medium amylose chains (DP ∼100–5000), which enhanced both the structure with short-range order and crystal structure within the gel matrix. However, after 200 min of freezing, water-holding capacity and viscoelasticity decreased, and the submicrostructure became more loosely packed. This was due to ice crystal growth, which caused extrusion and a reduction in amylose content, short-range ordered structure, and crystallinity within the starch gels (Chi et al., 2023). Precise control of freezing rate and temperature is crucial for preserving the structural integrity of starch gels by regulating the size and number of ice crystals. Rapid freezing generates numerous fine ice crystals, which helps maintain the starch gel network structure (Qiao & Peng, 2024). Research by Gong et al. (2020) demonstrated that rapid freezing at −40 °C and − 80 °C effectively delayed retrogradation in oat roll starch, prevented ice crystal enlargement and structural rupture, and maintained internal structural integrity. Notably, quick freezing at −80 °C showed superior results. However, extended storage time led to ice recrystallization, resulting in larger ice crystal size and decreased quantity.

#### 3.2.3 pH

The hydrolysis of starch occurs under acidic pH conditions, while an alkaline pH disrupts hydrogen bond interactions between starch molecules, enhancing the interaction between starch molecules and water. Therefore, the effects of pH on starch gel structure are attributed to both the hydrolysis and dissolution of starch (Jia et al., 2023). The changes in gel formation under acid-treated or alkali-treated conditions can be better understood by analyzing how starch molecules behave under these conditions. Chatpapamon and colleagues found that the heat-moisture treatment (HMT) of native cassava starch gel resulted in a transparent, viscous, firm, and elastic texture. HMT tapioca starch gel showed a white and cloudy appearance after being pickled at pH 3.0, and obtained similar non-stick consistency and spoon consistency as yoghurt. In contrast, the HMT cassava starch gel treated with pH 11.0 was brown, cloudy, stable, non-sticky and pudding-like (Chatpapamon et al., 2019). The effects of starch gel formation under acidic and alkaline conditions can be diametrically opposite. For pregelatinized and granulated corn starch swollen by cold water, textural parameters, turbidity, and freeze-thaw stability of the samples were decreased and at solubility was increased at pH 3.0 and pH 5.0. Conversely, the rheological and mechanical properties as well as freeze-thaw stability and turbidity of the starch paste were increased at pH 9.0 and pH 11.0 (Hedayati et al., 2016). In addition, the phosphorylation level of root starch and tuber starch is significantly high, with phosphate groups predominantly binding to the C-6 and C-3 positions of glucose residues in branched starch (Blennow et al., 2002). Research proved that pH can modulate the gel formation tendency of starch by influencing the interactions of phosphate groups. Notably, the phosphate groups attached to C-3 extend from the hydrophilic surface of the double helix, thereby affecting both double helix stacking and starch retrogradation (Blennow et al., 2002). The study conducted by Fang et al. (2020) examined the behavior of waxy potato starch, waxy corn starch, and waxy rice starch at various pH levels. In acidic conditions (pH 4.0-pH 6.0), the protonation of phosphomonoesters reduced electrostatic repulsion between molecules, leading to starch aggregation and the formation of a robust viscoelastic gel with high G′ and an opaque appearance. In alkaline conditions (pH 8.0-pH 10.0), however, waxy potato starch exhibited the opposite behavior, with diminished charge repulsion due to the high concentration of ionized phosphomonoesters hindering gel formation. It is noteworthy that under conditions of slight acidity or insufficient acidity for starch cleavage, hydrogen ions appear to facilitate the protonation of the hydroxyl groups on the starch. This may have the effect of attenuating the interactions between starch molecules and inhibiting the formation of the starch gel structure. Wang, Qin, et al. (2023) demonstrated that under acidic conditions (pH 3–7), as the pH of pea starch decreased, the starch gel structure became looser, the hardness and elasticity of the gel decreased, and hydrogen bonding weakened.

### 3.3 Food ingredients enhancing starch gel structure

#### 3.3.1 Salt ion-starch gels

Salt ions, a ubiquitous food ingredient, play dual roles in enhancing flavor and acting as preservatives to extend the shelf life of foods. The concentration and diversity of salt ions are pivotal factors that influence the formation of starch gels (Rostamabadi et al., 2023). The primary mechanisms through which the interaction between salt ions and starch impacts the formation of gel structure can be summarized as follows: (1) the influence of salt ions on the formation and disruption of water and starch structures; (2) the electrostatic interaction occurring between starch and salt ions (Li, Lv, Huo, Jia and Li, 2023). Structure-making (salt-out) ions with smaller sizes or lower polarization tend to stabilize the hydrogen bonds between starch molecules, thereby promoting starch retrogradation and enhancing starch gel strength. Conversely, structure-breaking (salt-in) ions with relatively larger diameters or less symmetrical structures exhibit a weaker tendency towards retrogradation (Li et al., 2015). The addition of structure-making ions (F^−^, SO_4_^2−^) increased the gel strength, G' and G" of potato starch. However, structure-breaking ions (Br^−^, NO^3−^, I^−^, SCN^−^, Na^+^) had the opposite effect on potato starch. The sequence of effects of salts on the retrogradation of potato starch generally as F^−^, SO_4_^2−^, Cl^−^, Br^−^, NO^3−^, I^−^, SCN^−^ for anions and K^+^, Na^+^, Li^+^ for cations, in accordance with the Hofmeister series (Chen et al., 2014). The formation of starch gel structure showed a significant correlation with the concentration of salt. Zheng et al. (2022) emphasized that low concentrations of NaCl solution (50 and 100 mM) significantly improved the gel strength by promoting amylose hydrogen chain polymerization in wheat starch. However, even when exposed to high concentrations of NaCl solution (150 mM), the gel maintained a soft and elastic texture after long-term storage. This phenomenon could be attributed to the hindrance of polymerization between starch molecules and the inhibition of crystal growth caused by the entry of Na^+^ ions into starch molecules. These findings underscore the significant impact of salts as essential seasoning agents for starch-based foods on starch gel formation.

#### 3.3.2 Protein-starch gels

Proteins are widely present in starch-based foods and play a crucial role in developing starch gel structure during processing and storage (Xu, Ji, et al., 2023). Numerous studies have demonstrated that proteins interact with starch by either weakening its hydrogen bonds or forming a protective barrier on its surface, thereby inhibiting starch expansion and impeding gel formation (Xu, Wang, et al., 2024). Conversely, certain proteins can form interpenetrating networks with starch during gelatinization, facilitating the formation of starch gels. These modifications in formulation can lead to unforeseen implications for the quality attributes of starch-based products (Wang, You, et al., 2023). The interactions between protein and starch can be classified into three types: (1) protein permeation and physical adsorption onto the surface of starch particles; (2) protein aggregation within continuous phases; and (3) covalent and non-covalent binding between starch molecules and proteins (Scott & Awika, 2023). The study conducted by Zhang, Wang, et al. (2019) demonstrated that incorporating rice protein promotes the formation of a more compact and uniform structure in rice starch gels while inhibiting its recrystallization. This phenomenon could be attributed to the steric hindrance induced by rice protein, which restricted intermolecular cross-linking among starch molecules. Additionally, the hydrophilic nature of rice protein hinders water transport and enhances water retention. The addition of rice protein exceeds 12 % was found to enhance the gel strength and grid (Wu et al., 2023). This was because spontaneous protein-protein interactions, primarily mediated through disulfide bonds, which replaced starch-protein interactions. Compared to corn starch alone, the incorporation of pea protein modifies the rheological properties of corn starch from elastic to more viscous. Conversely, the presence of pea protein in cassava gel led to the formation of a more structured and solid (low brown) gel. The lower gelatinization temperature of cassava gel facilitated interactions between starch chains and protein chains, resulting in a superior network structure (Ribotta et al., 2012). The enhanced gel formation ability between corn starch and salmon protein is attributed to an increased number of binding sites, facilitated by the presence of branched starch particles that serve as structural fillers. It led to improved strength and shape retention over time, evident from the gradual rise in both G' and G" during interaction between protein and starch (Carvajal-Mena et al., 2024). Yu et al. (2023) reported that incorporating rice glutelin enhanced both G' and G″ of extruded rice starch. This enhancement was due to increased protein content, which promoted a higher cross-linking density between rice glutelin and starch within the extrusion mixture. During the extrusion process, interactions between starch and gluten occur through both Maillard reactions and non-covalent bonds such as hydrogen bonding, hydrophobic interactions, and electrostatic interactions.

#### 3.3.3 Polysaccharide-starch gels

Polysaccharides are natural polymers that encompass a diverse range of bioactive constituents. They are widely used as additives in starch-based food products due to their excellent safety profiles, minimal toxicity, and high efficacy (Bello-Perez & Flores-Silva, 2023). Previous studies have demonstrated that the impacts of polysaccharides on the formation of starch gels can be either positive or negative, contingent upon the specific types of polysaccharides and starches involved. Table 4 showed a summary of existing research on how polysaccharides enhance the structural integrity of starch gels. The study conducted by Ren et al. (2020) demonstrated that during the gelatinization process of rice starch, leached amylose forms a network around starch granules through hydrogen bonds and electrostatic forces, interacting with *Mesona chinensis* polysaccharides. Consequently, *Mesona chinensis* polysaccharides contributed to increased hardness, gel strength, and water holding capacity while promoting a more compact and organized network structure. The concentration of polysaccharides also influences the formation of starch gel structure. A low concentration (≤1 g/100 g) of *Mesona chinensis* polysaccharides had a positive impacts on the development of debranched waxy maize starch gel networks, and excessive amounts (3 or 5 g/100 g) of *Mesona chinensis* polysaccharides could lead to self-aggregation and inhibited the organized rearrangement of amylose and, compromising the compactness and stability of the gel (Xiao et al., 2021). The enhancement was attributed to the synergistic effect of polysaccharides, leached amylose and starch particles. Simultaneously, the formation of a cross-linking structure by polysaccharides and amylose hinders the long-term retrogradation of starch gels (Li et al., 2023). The mechanism underlying the enhancement of gel structure may vary in different polysaccharide-starch systems. Certain polysaccharides primarily facilitate starch gel formation through hydrogen bondings. For instance, tremella polysaccharides interact with potato starch via hydrogen bonds (mainly) and weak electrostatic interactions, creating a compact gel structure (Yang, Pan, et al., 2022). Additionally, κ-carrageenan, konjac gum, and *Mesona chinensis* Benth polysaccharide facilitated the formation of cassava starch gels through hydrogen bonding interactions, resulting in increased G', G", gel strength, and hardness, and leading to a more stable and compact microstructure (Song et al., 2024). The addition of xanthan gum, sodium alginate, and guar gum also improved the rheological and structural properties, such as gel strength and elasticity, of lotus root starch, through the formation of hydrogen bonds (Han et al., 2024). In summary, the enhancement of gel structure by polysaccharides primarily occurs through two mechanisms: (1) the generation of a cross-linked structure via hydrogen bonding between starch and polysaccharide; and (2) the creation of a physical barrier on the surface of starch through electrostatic or hydrogen bonding interactions with polysaccharides.

## 4 Modified methods enhancing starch gel structure

Starch modification methods can be categorized into physical, chemical, and enzymatic modifications. To enhance starch gel structure, physical and enzymatic modifications leverage hydrogen bonding between starch molecules, while chemical modifications rely on chemical groups to enhance cross-linking among starch molecules (Obadi & Xu, 2021). Table 3 presents various modification methods for enhancing starch gel structure.

**Table 3:** Enhancement of starch gel structure by adding food ingredients.

Factors	Starch source	Conditions	Results	References
Salt ions	Potato starch	Structure-making ions (F^−^, SO_4_^2−^) Structure-breaking ions (Br^−^, NO^3−^, I^−^, SCN^−^, Na^+^)	Reduced the freeze-thaw stability and increased the gel strength, G' and G" of potato starch. Showed an opposite trend on potato starch gels compared with structure-making ions.	Chen et al., 2014
	Wheat starch	Low concentrations of NaCl solution (50 and 100 mM) The addition of high concentrations of NaCl solution (150 mM)	Significantly enhanced the gel strength. Maintained the gel a soft and elastic texture even after long-term storage.	Zheng et al., 2022
Proteins	Rice starch	Rice protein	Promoted the formation of a denser and more uniform structure in rice starch gels, while inhibiting its recrystallization.	Zhang, Wang, et al., 2019
	Indica rice starch	Rice protein exceeds 12 %	Gel strength and grid were enhanced.	Wu et al., 2023
	Cassava gel	Pea protein	Leading to the formation of a more structured and solid (low brown) gel.	Ribotta et al., 2012
	Corn starch	Salmon protein	Improved strength and shape retention over time, evident from the gradual rise in both G' and G".	Carvajal-Mena et al., 2024
	Extruded rice starch	Rice glutelin	Resulted in an improvement of the G' and G".	Yu et al., 2023
Polysaccharides	Rice starch	*Mesona chinensis polysaccharides*	Increased hardness, gel strength, and water holding capacity while promoting a more compact and organized network structure.	Ren et al., 2020
	Wheat starch	Low concentrations (≤1 g/100 g) *Mesona chinensis* polysaccharides	Positively impacts the development of debranched waxy maize starch gel networks.	Xiao et al., 2021
		Excessive amounts (3 or 5 g/100 g) *Mesona chinensis* polysaccharides	Decreased the compactness and stability of the gel.	
	Potato starch	Tremella polysaccharides	Formed a compact gel structure.	Yang, Du, et al., 2022
	Cassava starch	κ-carrageenan, konjac gum, and *Mesona chinensis* Benth polysaccharide	Increased G', G", gel strength, and hardness, and leading to a more stable and compact microstructure.	Song et al., 2024
	Lotus root starch	Xanthan gum, sodium alginate, and guar gum.	Gel strength and elasticity enhanced.	Han et al., 2024

**Table 4:** Enhancement of starch gel structure by modification.

Factors	Starch source	Conditions	Results	References
HMT	Potato starch	23.56 % moisture at 90 °C for 1.5 h.	Reduced starch pasting viscosity, enhanced thermal and shear stability, as well as gel strength and hardness, promoted the formation of starch gel structures,	Deng et al., 2022; Navaf et al., 2022; Sindhu et al., 2019
	Corypha Umbraculifera L. Starch	25 % moisture at 110 °C for 1 h.		
	Buckwheat starch	30 % moisture at 85, 120 °C for 6 h.		
PGT	Oat starch	Stirred in a 70 °C water bath for 10 min and dried using a spray dryer.	Rheological properties enhanced.	Shen et al., 2023
	Corn starch	Corn starch was suspended in distilled water (24 %, *w*/w) and extruded at 180 °C (Twin-screw extruder with a 0.5 mm nozzle diameter of and a screw speed of 260 rpm).	Gel strength enhanced.	Li et al., 2020
	Rice flour	Cassava starch was treated with ultrasound for 30 min at 75 °C at frequency of 2 × 10^4^ Hz and power 560 W.	PGS functions as an additive promotes the development of a resilient gel-like network structure.	Wang et al., 2019
DHT	Wheat starch	Processed by dry heating for 2 h and 4 h at 130 °C.	Enhanced static structural strength, external stress resistance, and gel hardness.	Maniglia et al., 2020
	Rice starch	Heated for 0, 2, 4 h at 130 °C.	Increased pasting viscosity and enhanced gel structure of rice starch.	Qiu et al., 2015
MT	Potato starch	Low microwave power and short treatment time (300 W for 1, 3, and 5 min) at 21.00 % moisture.	G', G", pasting temperature and pasting viscosity were increased and positively correlated with treatment time, moisture content decreased to 6.53 % at a treatment time of 5 min.	Kumar et al., 2020
	Quinoa starch	9 W/g power density for 20 s at 11.75 % moisture.	A more compact network structure was observed under the microstructure, with a decrease in moisture content to 10.59 % at a treatment time of 20 s.	Cao et al., 2022
	Potato starch	2450 MHz, 750 W, and a solid concentration of 33 % (*W*/W).	The impact on gel structure was manifested by an increase in G' and G" with increasing duration of MT from 0 to 15 s, followed by a subsequent decrease from 15 to 20 s.	Xie et al., 2013
UT	Corn starch; potato starch; pea starch	100–600 W for 5–35 min.	G' and G" increased and tan δ decreased.	Zhang et al., 2021
	Lotus starch	270 W for 30 min.	(1) G' and G" increased.	Wang, You, et al., 2023
		360 and 450 W for 30 min.	(2) Swelling power, pasting viscosity, G' and G" all reduced.	
	Pea starch	680 W for 10 and 20 min.	Pasting viscosity, transparency decreased and gel strength increased.	Falsafi et al., 2019
		680 W for 30 min.	Gel strength decreased.	
Cross-linking modification	Rice starch	Lactic acid and citric acid (20 % and 40 % *w*/*v* concentration) combined with heat-moisture treatment.	(1) Lactic acid starch ester and citric acid starch ester showed that G' increased, and G' > G”, reflecting viscoelasticity. (2) The G' of citric acid starch ester was slightly higher than that of lactic acid starch ester.	Butt et al., 2021
	Corn starch	Citric acid (w/w, on dry starch) combined with Microwave treatment.	Enhanced the freeze-thaw stability of starch by forming a cross-linked structure.	Hu et al., 2021
	Waxy wheat starch, waxy maize starch, and waxy tapioca starch	STMP/STPP (99:1) at different levels (0.01 %, 0.05 %, and 0.1 %) and	Less easily disintegrated in the gelatinization process and enhancing the gel strength.	Gu et al., 2024
	Barnyard millet starch	STMP at different levels (1, 3 and 5 %).		Sharma et al., 2021
Two commercial amylases (A- and N-amylase) teatment	Cross-linked tapioca starch	A- or N-amylase (1.53 U/g and 6.64 U/g, respectively) was added reacting at 50 °C on a water bath for 2, 8, and 23 h.	(1) A-amylase increased the G' of cross-linked starch gels by approximately 10 % under similar degrees of hydrolysis.	Yuan, Wang, Bai and Birte, 2022
			(2) N-amylase produced a solid gel within 15 min and increased the G' of the starch gels by nearly 30 % after 23 h of hydrolysis.	
α-amylase from Aspergillus Niger	Cassava starch	α-amylase (3 g) for 23 h at 50 °C.	Improved the elasticity of starch gel by constructing a strong filler-in-matrix type structure.	Ichihara et al., 2016
Pululanase teatment	Acorn starch	48 U/g for 2 h, 6 h, 10 h, 14 h, and 18 h at 55 °C.	Enhanced gel strength.	Chen et al., 2022
Transglucosidase treatment	Pullulanase-treated rice starch	Treated with transglucosidase (1650 U/g) at 55 °C for 6, 12, 18, 24 h.	Formed a tighter three-dimensional gel network structure with higher hardness and springiness.	Geng et al., 2024
Induced electric field-pullulanase treatment	Corn starch	Treated with pullulanase under induced electric fields (50 V, 75 V, and 100 V).	The gel strength was improved by induced electric field-pullulanase treatment.	Liang et al., 2024
Pullulanase-treated starch mixed with xanthan gum or sodium alginate	Pea starch	Treated with pullulanase (0.3 U/mg) at 58 °C for 12 h and mixed with xanthan gum or sodium alginate (0.2 %、0.5 % and 1.0 %).	The addition of xanthan gum or sodium alginate enhanced the rheological properties.	Liu et al., 2023

### 4.1 Physical modification

The mechanisms underlying physical modification that enhance the gel structure of starch primarily involve disrupting hydrogen bonds between starch molecules, fragmenting crystalline regions within starch, altering the amylopectin-to-amylose ratio, cleaving or aggregating molecular chains, and reorganizing starch molecules (Ye & Baik, 2023). Physical modification of starch can be safely applied in food applications.

#### 4.1.1 Heat-moisture treatment

The process of HMT involves subjecting starch particles to temperatures above their glass transition temperature for 1–24 h. This hydrothermal treatment is conducted at relatively low water content (<35 %) and higher temperatures (80–140 °C) (Wang et al., 2021). During the HMT process, starch is sealed and high pressure is applied to prevent evaporation and maintain moisture levels. The energy of excess water molecules is converted into kinetic energy, resulting in extensive fragment movement and alterations in the internal structure of the starch. HMT enhances the internal structure by increasing fluidity within starch chains and helical structures under conditions of limited moisture and elevated temperatures, thereby improving stability through modifying or reorganizing the internal structure (Schafranski et al., 2021). It has a significant impact on the formation of starch gels. Studies found that HMT effectively promoted the formation of starch gel structures. HMT also decreased the viscosity of starch paste while improving its thermal stability, shear stability, gel strength, and hardness (Deng et al., 2022; Navaf et al., 2022; Sindhu et al., 2019). It could be attributed to the recomposition of starch chains, resulting in increased intermolecular interactions that enhance paste stability and gel properties. Such conclusions were supported by studies on HMT of potato starch (Deng et al., 2022), Corypha Umbraculifera L. starch (Navaf et al., 2022), buckwheat starch (Sindhu et al., 2019). However, some studies have found that for certain starches, HMT can cause thermal degradation of starch molecules, resulting in restricted particle damage and swelling. One study reported that HMT significantly reduced the cross-linking degree of four types of pea starch, lowered gel hardness, and weakened the ability to form starch gel structure (Cheng et al., 2024). Similar downward trends were observed in previous research on the HMT of amaranth starch (Siwatch et al., 2022).

#### 4.1.2 Pre-gelatinized treatment

Pre-gelatinized starch (PGS) is a type of cold-water soluble starch that undergoes gelatinization through heating and rapid drying at high temperatures, transforming its original β-structure to an α-structure. Thus, PGS is also referred to as α-starch (Ma, Zhu, et al., 2022). During the pre-gelatinization process, water molecules disrupt the hydrogen bonds of starch molecules, destroying the crystalline structure of starch granules. This leads to swelling in water and the formation of a gel network with viscoelastic properties through covalent interactions, producing a starch paste with good dispersion and thickening stability (Ma, Liu, et al., 2022; Ye & Baik, 2023). PGS exhibits high water absorption, gelatinization degree, and viscoelasticity, enhancing elasticity and moldability of food products. It also functions as a binder during food processing (Lee & Yoo, 2023). Rheological improvements in oat starch were observed following pre-gelatinization treatment (PGT) via spray drying, which was attributed to the disruption of glycosidic bonds, resulting in a decrease in the molecular weight of amylopectin and amylose, and an increase in short linear chains (Shen et al., 2023). Similarly, the PGT of corn starch significantly enhanced gel strength, with the gel strength of drum-dried corn starch being 1.67 times that of extrusion-cooked corn starch (Li, Liu, et al., 2020). Additionally, PGS functions as an additive, facilitating the interaction among other starches and promoting the formation of a well-structured starch gel. In the case of glutinous rice flour, PGS was found to promote the development of a resilient gel-like network by influencing starch granules' behavior (Wang et al., 2019).

#### 4.1.3 Dry-heat treatment

Dry-heat treatment (DHT) is a heat treatment method similar to HMT, but it begins with drying starch (typically around 10 %) before applying high-temperature heat without sealing. This process results in an anhydrous or nearly anhydrous state with a water content of less than 1 %. During DHT, water loss and high temperatures lead to the rearrangement of starch chains (Liu, Zhao, et al., 2022). A DHT experiment conducted by Maniglia et al. (2020) found that DHT increased the static structural strength, external stress resistance and gel hardness of wheat starch. This improvement resulted from DHT-induced molecular depolymerization, which enhanced the recombination and packaging of starch molecules, thereby forming a more robust network structure. Similarly, DHT increased the pasting viscosity and enhanced the gel structure of rice starch through recrystallization (Qiu et al., 2015). Appropriate high-temperature processing for a specific duration can enhance the gel strength of high amylose rice starch and facilitate its gel formation. However, a study on high amylose rice starch found that temperatures above 110 °C for more than 1 h degraded the starch chains, resulting in a more fragile gel structure (Oh et al., 2018). Repeated DHT was reported to decrease the viscoelasticity, gel strength, and hardness of chestnut starch compared to native starch (Liu et al., 2020). This suggests that DHT does not support the formation of chestnut starch gels, particularly with prolonged treatment. The reduction in effective water content and concentration per unit volume caused by DHT was noted to affect the swelling and water absorption properties of starch particles, ultimately influencing the formation of the gel network structure (Liu et al., 2020).

#### 4.1.4 Microwave treatment

Microwave treatment (MT) produces thermal effects by heating polarizable molecules, such as water molecules and ions, when exposed to oscillating microwaves within electromagnetic fields (300–300,000 MHz). This heating occurs due to molecular rotation, friction, and collision (Oyeyinka et al., 2021). The previous study conducted by Kumar et al. (2020) revealed that applying low microwave power and short treatment times (300 W for 1, 3, and 5 min) to potato starch increased G′, G″, and pasting viscosity, while reducing moisture content from 21.00 % to 6.53 % after 5 min of treatment. These parameters showed a positive correlation with treatment duration, indicating excellent gel formation performance. This effect resulted from the gelatinization and cross-linking of molecular chains released from starch granules during microwave treatment. Cao et al. (2022) showed that MT at a power density of 9 W/g for 20 s dispersed the vesicular structure of quinoa starch aggregates, leading to the individualization of starch granules and improved hydration of starch and water molecules. Microstructural analysis revealed a more compact network structure with moisture content decreasing from 11.75 % to 10.59 %. However, excessive MT led to partial gelatinization of starch and the formation of a rigid structure. Numerous studies have shown that only moderate MT can enhance the gel structure of starch. For example, at 2450 MHz, 750 W, and a solid concentration of 33 % (*W*/W), the molecular weight of potato starch gradually decreased after MT for 5–15 s, with a sharp decline observed after 15–20 s. The impact on gel structure was indicated by an increase in G′ and G″ with increasing MT duration from 0 to 15 s, followed by a decrease from 15 to 20 s (Xie et al., 2013). In the study conducted by Zhong et al. (2021), amylose and amylopectin were treated separately by microwave. Prolonged MT (800 W, 3–8 min, 30 % moisture) reduced the G′ and G″ of both amylose and amylopectin due to starch molecule degradation, leading to a diminished capacity for gel network formation in excessively degraded molecules. In summary, microwave power and treatment time are crucial in regulating gel structure. Furthermore, water plays a critical role by influencing the dielectric properties of starch, which are highly controllable (Tao et al., 2020). Achieving the ideal gel structure requires adjusting the water content during MT. Although this aspect is less studied, it warrants further exploration (Li, Hu, Wang and Zheng, 2019).

#### 4.1.5 Ultrasonic treatment

Ultrasonic treatment (UT) enhances the movement of starch molecular chains through mechanical, cavitation, and thermal effects. The cavitation force generated by ultrasound disrupts starch structure, partially degrades starch chains, increases amylose content, and thereby enhances the starch gel structure (Zhang et al., 2021). UT applied to corn starch, potato starch, and pea starch at 100–600 W for 5–35 min resulted in increased G′ and G″ and decreased tan δ (Zhang et al., 2021). Similarly, the G′ and G″ of lotus starch treated at 270 W were higher than those of native starch (Wang, Qin, et al., 2023). However, high-power UT (360 and 450 W) led to the breakdown of lotus starch granules, destruction of the amorphous regions, and partial destruction of crystalline regions, resulting in reduced swelling power, pasting viscosity, G′, and G″. The strong mechanical force from the destruction of starch chains weakened the network structure strength (Wang, Liu, et al., 2023). Similar results were observed in a study by Falsafi et al. (2019) on oat starch. The duration of UT also significantly influences starch gel properties. For instance, pea starch subjected to UT at 680 W for 10, 20, and 30 min showed enhanced starch interactions due to short-chain molecules produced by ultrasound. This enhancement was indicated by decreased pasting viscosity, reduced starch paste transparency, and increased gel strength. After 30 min of treatment, excessive degradation of starch molecular chains led to a decrease in gel strength (Li, Ge, Guo and Liu, 2023). Therefore, appropriate UT can enhance starch gel structure, but excessively high ultrasonic power and prolonged UT can lead to excessive degradation of starch chains and weaken the gel network.

### 4.2 Chemical cross-linking modification

The numerous hydroxyl groups in starch molecules facilitate the incorporation of crosslinking agents, thereby enhancing intermolecular forces and improving the gel-forming ability of starch (Punia Bangar et al., 2024).

#### 4.2.1 Esterification cross-linking

Many carboxyl groups in organic acids were esterified with hydroxyl groups on starch under certain conditions to form cross-linked structure, which has obvious steric hindrance to the enzymatic hydrolysis by amylase. Previous studies have shown that organic acid starch esters have good anti-digestion characteristics, making them suitable for functional foods (Karma et al., 2022). The preparation of organic acid starch esters usually requires the combination of other thermophysical modification methods, as high temperatures are more conducive to forming ester bonds. Butt et al. (2021) used lactic acid and citric acid (at 20 % and 40 % *w*/*v* concentrations) combined with HMT to prepare lactic acid and citric acid starch esters from rice starch. Rheological results showed that increased G′ and G′ > G″, reflecting improved viscoelasticity. This enhancement was due to the enhancing of the rice starch gel structure through cross-linking and esterification by lactic and citric acids. The G′ of citric acid starch ester was slightly higher than that of lactic acid starch ester, likely due to citric acid's three hydroxyl groups, which facilitate stronger cross-linking and a more robust gel network. Similarly, citric acid corn starch ester prepared with microwave assistance improved freeze-thaw stability by forming a cross-linked structure (Hu et al., 2021). These findings suggest that organic acids with multiple hydroxyl groups can effectively cross-link starch, enhancing the gel properties of the cross-linked starch.

#### 4.2.2 Other chemical cross-linking modification

The effects of cross-linking modification on the properties of starch are remarkably significant, as even minimal cross-linking between starch molecules can lead to substantial changes in gelatinization and gel formation (Radi et al., 2022). Cross-linked starch is obtained by the reaction of alcohol hydroxyl groups on starch molecules with multifunctional compounds, resulting in chemical bonds between starch molecules (Punia Bangar et al., 2024). Common cross-linking agents include phosphoryl chloride, adipic acid diethyl ester anhydride, sodium trimetaphosphate (STMP), and mixtures of STMP with sodium tripolyphosphate (STPP). All these agents are FDA-approved for food-grade applications (Obadi & Xu, 2021). These chemical bonds act as bridges between starch molecules, inhibiting the gelatinization and disintegration of starch particles while improving the shear strength and structural stability of starch gels. Cross-linking groups specifically reinforce both intermolecular and intramolecular interactions within starch molecules, preventing the extension of starch chains (Bodjrenou et al., 2023). This restricted swelling behavior reduces component leaching, thus preventing the formation of a fully developed “filler in matrix” gel network. Consequently, it significantly enhances the stability of the food during high-temperature processing. These effects were observed in experiments where Gu et al. (2024) cross-linked waxy wheat, maize, and tapioca starch with STMP/STPP (99:1) at varying levels (0.01 %, 0.05 %, and 0.1 %), and Sharma et al. (2021) prepared cross-linked barnyard millet starch using STMP at different concentrations (1 %, 3 %, and 5 %). However, excessive cross-linking can negatively impact starch gel formation. High degrees of cross-linking restrict the stretching of starch molecules, limit swelling behavior, and significantly reduce pasting viscosity (Kou et al., 2022). Therefore, it is crucial for researchers to carefully select reaction conditions to control the degree of starch cross-linking.

### 4.3 Enzymatic modification

In the food industry, enzymatic reactions involving starch exhibit specificity and mildness. Compared to physical and chemical modifications, these reactions yield fewer by-products while ensuring stable safety and quality. Researchers have explored various enzymatic modification techniques aimed at altering starch properties for novel applications in the food industry (Zhang, Chen, et al., 2019). The principle of enzymatic modification involves hydrolyzing starch to alter its molecular weight, amylose/amylopectin ratio, and amylopectin chain structure. This modification enhances the starch's application value and effectiveness (Punia Bangar et al., 2022). In our summary above, more amylose and small molecule amylopectin contribute to the formation of starch gel structure. α-amylase is a common starch-degrading enzymes. As an *endo*-enzyme, it randomly hydrolyzes α-1,4 glycosidic bonds (Mendonça et al., 2023). Yuan et al. (2022) found that two commercial α-amylases, A-amylase and N-amylase, hydrolyze cross-linked tapioca starch based on different patterns. A-amylase increased the G' of cross-linked starch gels by approximately 10 % under similar degrees of hydrolysis. In contrast, N-amylase formed a solid gel within 15 min and increased the G' of the starch gels by nearly 30 % after 23 h of hydrolysis. A-amylase primarily reinforces the gel network structure by breaking down the amorphous regions of cross-linked starch particles and generating high amylose content. N-amylase, however, breaks down both amorphous regions and thin layers within crystalline regions, producing smaller amylose and amylopectin. It forms more “filler in matrix” units, resulting in a more compact gel structure. Similar to N-amylase, Ichihara et al. (2016) proved that α-amylase from *Aspergillus niger* treatment improved the elasticity of starch gel by constructing a strong filler-in-matrix-type structure. Pululanase can directly hydrolyze the α-1, 6-glucoside bond in amylopectin, and this debranching process will produce more linear amylose, Chen et al. (2022) used pululanase to hydrolyze acorn starch, which improved the starch chain mobility and ordered arrangement of starch chain, and thereby improve gel strength. However, enzymatic hydrolysis is easy to produce an excess of starch molecules that reduce cross-linking ability, thereby impairing the gel formation capacity of starch (Xie et al., 2022). For example, maltogenic α-amylase treatment effectively reduced the molecular weight and chain length of rice starch, which greatly reduced the cross-linking ability between starches, resulting in diminished elastic properties such as gel strength and creep (Wang, Bai, et al., 2022). Similarly, β-amylase hydrolysis led to decreased G′ and G″ in wheat starch (Li et al., 2023). Excessive enzymatic hydrolysis can produce too many hydrolysates, negatively affecting starch gel formation. To address this, Geng et al. (2024) employed transglucosidase treatment on pullulanase-treated rice starch. This treatment connected some short chains to longer linear chains via α-1,6-glucosidic bonds, forming structures with fewer branches. The shorter branched chains enhanced interactions between starch molecules, resulting in a tighter gel network with increased hardness and elasticity.

In addition, the processing conditions for enzymatically modified starch significantly influenced starch gel formation. During the enzymatic degradation of starch, it is crucial to maintain an optimal pH and temperature to sustain amylase activity. Deviations in pH, whether too low or too high, inactivated the enzyme, while high temperatures caused denaturation, and low temperatures inhibited enzyme activity (Negi et al., 2024). Furthermore, certain food ingredients in enzyme-starch system acted as natural enzyme inhibitors to inhibit enzymatic degradation. According to a study by Peng et al. (2024), catechol, quercetin and hesperidin interacted with α-amylase through non-covalent bonds, affecting the enzymatic hydrolysis of starch. Similarly, gluten significantly reduced the activities of α-amylase and glucosidase (Yu et al., 2024). On the other hand, specific pretreatment methods can affect enzyme degradation by changing the molecular structure of starch. Geng et al. (2023) reported that the combined modification of preheating and pullulanase treatment released more short amylose and reduced starch interactions, thus weakening the gel network. What's more, Liu et al. (2023) found that high hydrostatic pressure created an intermediate state between semi-crystalline and granular starch paste, increasing the sensitivity of corn starch to maltogenic α-amylase, resulting in a rich content of short-chain pectin starch. Another study showed that the in vitro enzymatic hydrolysis of rice starch with pullulanase under induced electric fields (50 V, 75 V, and 100 V) progressively increased the amylose content and gel strength of the starch samples (Liang et al., 2024). These findings indicate that different pretreatment conditions distinctly affect starch gel formation, likely due to variations in enzymatic hydrolysis levels. Therefore, in order to obtain a stronger starch gel structure, it is necessary to further explore the experimental conditions of enzyme treatment or regulate the formation of gel by adding edible agents. For example, pullulanase treatment of pea starch generated a high amylose content, and the addition of xanthan gum or sodium alginate further supported gel formation through interactions with the amylose (Liu et al., 2023).

## 5 Applications of treatments based on enhancing starch gel structure in foods

### 5.1 Starch-based foods product quality

**Fig. 2:**
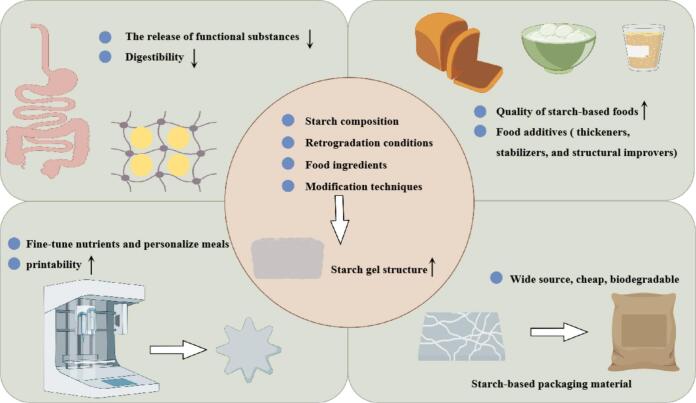
Application of enhancing starch gel structure in foods.

The gel network structure is crucial for the quality of starch-based foods, and its enhancement can substantially improve product attributes (Fig. 2). Pre-gelatinized starch (PGS) has emerged as a viable substitute for gluten in dough formulations, facilitating the formation of a cohesive network through interactions with other starches. For instance, PGS types such as drum-dried tapioca starch and extrusion-cooked maize starch had been shown to significantly enhance the tensile strength and textural properties of Tartary buckwheat noodle dough sheets (Obadi et al., 2020). The incorporation of egg white improved the firmness, chewiness, elasticity, shear resistance, and water retention capacity of steamed cold noodles (a wheat starch-based gel food), thereby contributing to the advancement of novel gluten-free food products (Bai et al., 2022). Additionally, rapid freezing and storage at −18 °C could effectively inhibit starch retrogradation, delay the destruction of gel structure by ice crystals, and preserved the textural properties of cooked rice for at least 7 months (Yu et al., 2010). Reyniers et al. (2020) found that the crispness of potato chips, where potato starch is the primary ingredient, can be modulated by the length of amylose chains. Treatment with α-amylase from *Bacillus stearothermophilus* before dough preparation enhances the starch's strength, resulting in chips with a denser structure and crispier texture. Furthermore, modified starches with altered gel properties serve as effective additives to improve food quality. For example, cross-linked starch is commonly utilized as a thickening agent in various food products, including soups, sauces, gravies, breads, and dairy items, due to its ability to stabilize food systems (Punia Bangar et al., 2024; Wei, Wu, Ren, Yu, & Sun, 2024).

### 5.2 Starch-based foods nutritional quality

Long-term hyperglycemia and unstable blood sugar levels are associated with chronic conditions such as diabetes, hypertension, and obesity (Yang et al., 2023). As a crucial energy source, starch-based foods with low digestibility have become a focus of research. Studies suggest a significant correlation between the strength of starch gel networks and starch digestibility (Fig. 2). Starch granules in foods with denser network structures exhibit reduced expansion during cooking. This dense network acts as a barrier to starch-degrading enzymes, thereby decreasing enzymatic digestion post-consumption (Jia et al., 2023). During food processing, starch gels with higher gel strength often result from increased levels of linear chain starch. These starches contribute to the formation of more crystalline regions during gelation, which reduces the digestibility of foods rich in linear starch content. Consequently, selecting raw materials with a high proportion of linear starch or employing suitable pretreatments to enhance linear starch content and enhance starch gel networks is an effective strategy for producing low glycemic index (GI) foods. For instance, in a study by Ouyang et al. (2024), among three rice varieties, the rice with high linear starch content exhibited the highest resistant starch levels. Similarly, Shah et al. (2023) reported that ultrasound treatment during the soaking process of parboiled rice promoted linear starch formation, resulting in a denser structure and increased resistant starch content. Additionally, specific macromolecules can interact with starch via cross-linking, resulting in stronger network structures and greater spatial hindrance to starch-degrading enzymes. In oat dough, the cross-linked network formed between oat protein and oat starch demonstrated increased G' and G", effectively acting as a barrier to enzymatic digestion (Sun et al., 2023). Similar effects were observed with soy protein isolate and whey protein isolate on wheat starch (Zhang et al., 2023). Polysaccharides can also enhance starch's mesh-like structure, creating a barrier between starch and digestive enzymes. The interaction between potato starch and tamarind polysaccharides resulted in a stable gel structure, reducing enzyme-starch contact (Yang, Du, et al., 2022). β-glucan reduced bread GI and α-amylase activity by intertwining with starch to form a more stable gel network (Hu et al., 2022). By appropriately processing and incorporating exogenous substances, the gel structure of starch can be enhanced, leading to the development of functional starch-based foods with reduced digestibility.

Starch-based gels have been extensively studied as carriers of functional ingredients in food (Fig. 2). Certain bioactive compounds, such as curcumin, β-carotene, and anthocyanins, are susceptible to degradation due to light, heat, oxidation, and decomposition. The cross-linking between starch molecules or between starch and other macromolecules forms the network structure, and the functional components are uniformly wrapped in the network. This encapsulation slows their release and improves their stability and bioavailability. For example:

Acetylated cassava starch hydrogels (Meng et al., 2020): Derived from cassava starch and modified with maleic anhydride, these hydrogels exhibit excellent pH sensitivity. At a degree of substitution of 0.250, the adsorption capacity of the starch-based hydrogel is 399.23 μg/g, with an encapsulation efficiency of 80 % for curcumin.

Sodium hexametaphosphate-crosslinked starch aerogels (Zhang, Wang, Liu, Wu and Ouyang, 2023): Prepared using sodium hexametaphosphate as a cross-linking agent, these starch-based aerogels served as carriers for β-carotene. In simulated in vitro digestion, β-carotene was released rapidly within the first 60 min. After 48 h of UV exposure and 4 weeks of room temperature light exposure, the retention rate of β-carotene remained significantly higher (27.1 % and 37.8 %, respectively) compared to non-encapsulated β-carotene.

Carboxymethyl starch and chitosan hydrochloride cross-linked gels (Li et al., 2019): These gels, formed through amide bonds, exhibited high viscoelasticity. They served as carriers for curcumin, with pH sensitivity and substantial encapsulation efficiency (ranging from 89.49 % to 94.01 %).

In summary, starch gels have been extensively validated as efficient carrier systems for enhancing the stability and facilitating the controlled release of functional ingredients in food applications.

### 5.3 Starch-based packaging materials

The practical use of food packaging materials requires excellent mechanical properties (Fig. 2). However, compared to traditional plastics, starch-based biodegradable packaging has significant disadvantages due to its inferior mechanical properties, which hinder its application in food and product packaging (Lauer & Smith, 2020). Enhancing the starch gel structure is crucial for improving the mechanical properties of starch-based packaging materials. Previous studies have demonstrated that the tensile strength of amylose and starch-based films is positively correlated with the elongation at break. This finding aligns with our earlier conclusion that amylose significantly contributes to the formation of a more robust gel structure (Liu, Pan, et al., 2022). The tensile strength and elongation at break of corn, rice, and wheat starch-based films prepared by pullulanase debranching treatment were increased. This improvement was primarily due to the increase of amylose content resulting from starch debranching (Tang et al., 2022). Cheng, Chen, et al. (2023) found that the mechanical strength of pure native corn starch films and pullulan/native corn starch films surpasses that of pure waxy corn starch films and pullulan/waxy corn starch films, primarily due to the higher amylose content in native corn starch. Cross-linking modification of starch significantly enhances the gel structure and is a common method for preparing starch-based films. A study by Chi et al. (2024) reported that incorporating 5 % citric acid into cassava starch-based films significantly improved both tensile strength and elastic modulus while effectively reducing elongation. Specifically, the tensile strength was more than twice that of natural starch films. This enhancement was due to the cross-linking of starch molecules in the film, which improved the structural integrity of the starch gel and resulted in superior tensile strength. Additionally, incorporating macromolecules to enhance the cross-linking network of starch gel is a primary approach for preparing starch-based films. The composite film formed by *Zizania latifolia* polysaccharide with antioxidant activity and corn starch exhibited compatibility in terms of microscopic structure (Huang et al., 2023). The results revealed that the addition of *Zizania latifolia* polysaccharide significantly increased the swelling degree and elongation of the film, with the tensile strength of the film being highest at a *Zizania latifolia* polysaccharide concentration of 4 %. Furthermore, the synergistic utilization of diverse methodologies contributes to the enhancement of film preparation. Li et al. (2024) prepared films using starch/pectin biopolymers as the matrix, chitosan as the copolymer, and tartaric acid/citric acid as natural plasticizers. They fabricated bioplastic films through covalent ester/amide bonds between carboxyl and hydroxyl/amine groups in the cross-linked network, along with hydrogen bond interactions. As research advances, starch-based films are progressively evolving towards active packaging and exhibit significant potential for applications in advanced intelligent packaging engineering, including antimicrobial properties, antioxidant capabilities, and food freshness detection (Lauer & Smith, 2020). For example: Li et al. (2023) encapsulated curcumin in a cross-linked matrix composed of dialdehyde starch and gelatin, which was then incorporated into chitosan to prepare a composite film. This composite film blocked 94.48 % of UV radiation, achieved a free radical scavenging rate of 99 %, and exhibited inhibition zones of 21.4 mm and 19.9 mm against *Escherichia coli* and *Staphylococcus aureus*, respectively. Yang et al. (2024) utilized β-cyclodextrin to encapsulate betanin and incorporated berberine with aggregate-induced luminescence effects into the corn amylose biopolymer matrix. This intelligent film exhibited excellent responsiveness to volatile ammonia (0.025–25 mg/mL) and demonstrated recyclability for at least four cycles. It also facilitated macro-dynamic monitoring of shrimp freshness under daylight (red to yellow) and UV light (yellow-green to blue-green) at storage temperatures of 25 °C, 4 °C, and − 20 °C.

### 5.4 3D printing

The investigation of customized and diversified food production has become a prominent area of research. Three-dimensional (3D) food printing, an innovative high-tech application, utilizes computer-controlled laser or inkjet equipment to digitally design and manufacture 3D food objects. This technology enables precise regulation of nutritional content and personalized customization of meals, offering significant potential for tailored dietary solutions (Rong et al., 2023). The utilization of starch as a valuable substance in developing edible bio-inks for 3D food printing applications enables the creation of a diverse range of food products (Chen, Hu, et al., 2024). Starch 3D printability is closely related to its gel structure, and a certain gel strength can maintain the structural stability of the printed product (Fig. 2). However, most starches are not suitable for 3D printing directly. Low concentration of starch pasting will lead to decrease of strength and viscosity, which can not meet the requirements of 3D printing (Carvajal-Mena et al., 2024). Wheat starch gel derived from DHT exhibited reduced apparent viscosity and enhanced static structural strength during gelatinization. Additionally, wheat starch showed significant improvements in printability and reproducibility when subjected to DHT, particularly with a duration of 4 h (Maniglia et al., 2020). The combination of ultrasound treatment (UT) and microwave treatment (MT, 80 W) was also found to enhance the G′ and G″ values in a wheat starch-papaya system, which contributed to better shape retention and significantly improved printing accuracy (Xu, Zhang and Bhandari, 2020). Incorporating additional food ingredients is considered the most straightforward approach and has been the most studied in recent years. For instance, the combination of potato starch with 0.8 % xanthan gum and 0.2 % locust bean gum significantly increased the gel system's resistance to compression deformation while achieving a smooth, fine texture (Yu et al., 2022). Wedamulla et al. (2023) reported that pectin addition increased the 3D printability and enhanced the starch gel structure at higher temperatures (80 °C and 90 °C). Corn starch and salmon protein isolate gels exhibited viscoelastic behavior at different concentrations, with a predominant elastic component (G′ > G″), demonstrating the material's capability to flow as an independent filament within 3D structures (Carvajal-Mena et al., 2024). However, the gel structure is too strong to the printing gel extrusion, reducing the printing height and accuracy, so it is necessary to moderate the starch gel structure control (Zheng et al., 2023).

## 6 Conclusions and future perspectives

The formation of starch gels is a critical aspect of food processing, with significant implications for improving the performance, stability, and nutritional value of foods. Starch (comprising amylose and amylopectin) undergoes gelatinization upon heating in water, forming a paste, and reorders into a gel structure upon cooling. The gel structure of starch can be effectively improved by regulating certain internal and external factors. Existing research has shown that the presence of amylose and short-chain amylopectin results in more molecular entanglements, promoting the formation of the gel structure. Additionally, the presence of starch-related proteins, lipids, and phosphate groups, though minimal, significantly affects starch gel formation. Researchers have explored numerous methods to enhance the struture of starch gels. This includes controlling gelatinization temperature, storage temperature, and pH during starch gel formation, or adding salt ions to promote interactions between starch molecules. Certain macromolecular polysaccharides and proteins can enhance the network structure by forming an interpenetrating network with starch.

It is worth noting that starch gel structure enhancement by physical, chemical and enzymatic modification techniques has been widely studied. The physical modification of starch has the least impact on the environment, but it face the cost challenge to meet the industrial demand. Chemical cross-linking methods significantly improve gel structure with high efficiency and low cost, though they carry potential risks of chemical contamination and safety concerns. Enzymatic modification, on the other hand, offers a faster processing time and minimal environmental impact but is hindered by higher costs, making large-scale application less economically feasible.

Although starch gel structure has been extensively studied, there are still many issues in the future research and application of Although starch gel structure has been extensively studied, there are still many issues in the future research and application of starch gel struture enhancement that need further investigation and discussion: that need further investigation and discussion:

• Starch-based foods are typically multi-component systems, and the synergistic or antagonistic effects of these components on gel formation have not been thoroughly investigated. Further research is needed to comprehensively understand these interactions and optimize gel structure in complex food matrices.
• The effectiveness of starch gel modification methods under varying processing conditions and techniques remains uncertain. It is necessary to assess whether these modifications maintain their efficacy in practical applications.
• Excessive enhancement of starch gel structure can negatively impact food applications in several ways: (1) It may impair processing performance, appearance, and texture quality in certain foods. Lowithun et al. (2024) observed that while small particles with a broad size distribution and short-chain amylopectin contributed to gel enhancement, an overabundance of these elements led to gel rigidity and brittleness. (2) In starch-based films, excessive gel reinforcement increases stiffness and reduces membrane fluidity, as noted by Wang et al. (2022). (3) In 3D printing processes, moderate enhancement of starch gel improves product precision, but excessive gel strength can obstruct gel extrusion through the nozzle (Zheng et al., 2024).
• Although extensive research has been conducted on enhancing starch gel structures, practical integration of these advancements into food products and processing techniques remains an area in need of deeper exploration.

## CRediT authorship contribution statement

**Yongqiang Gong:** Writing – review & editing, Writing – original draft. **Shuzhi Xiao:** Supervision. **Zihan Yao:** Resources, Investigation. **Hongjie Deng:** Project administration, Formal analysis. **Xuan Chen:** Software. **Tao Yang:** Writing – review & editing.

## Declaration of competing interest

Authors declare no conflict of interest.

## Data Availability

No data was used for the research described in the article.

## References

[bb0005] Adamczyk G., Krystyjan M., Kuźniar P., Kowalczewski P.Ł., Bobel I. (2022). An insight into pasting and rheological behavior of potato starch pastes and gels with whole and ground chia seeds. Gels.

[bb0010] Bai J., Dong M., Li J., Tian L., Xiong D., Jia J., Yang L., Liu X., Duan X. (2022). Effects of egg white on physicochemical and functional characteristics of steamed cold noodles (a wheat starch gel food). LWT.

[bb0015] Bello-Perez L.A., Flores-Silva P.C. (2023). Interaction between starch and dietary compounds: New findings and perspectives to produce functional foods. Food Research International.

[bb0020] Bertoft E., Annor G.A., Shen X., Rumpagaporn P., Seetharaman K., Hamaker B.R. (2016). Small differences in amylopectin fine structure may explain large functional differences of starch. Carbohydrate Polymers.

[bb0025] Blennow A., Nielsen T.H., Baunsgaard L., Mikkelsen R., Engelsen S.B. (2002). Starch phosphorylation: A new front line in starch research. Trends in Plant Science.

[bb0030] Bodjrenou D.M., Huang Z., Liu T., Zheng B., Zeng H. (2023). Effects of crosslinking with sodium trimetaphosphate on structural, physicochemical, rheological and in vitro digestibility properties of purple sweet potato starch. Food Research International.

[bb0035] Butt N.A., Ali T.M., Moin A., Hasnain A. (2021). Comparative study on morphological, rheological and functional characteristics of extruded rice starch citrates and lactates. International Journal of Biological Macromolecules.

[bb0040] Cao H., Sun R., Liu Y., Wang X., Guan X., Huang K., Zhang Y. (2022). Appropriate microwave improved the texture properties of quinoa due to starch gelatinization from the destructed cyptomere structure. Food Chemistry: X.

[bb0045] Carvajal-Mena N., Tabilo-Munizaga G., Pérez-Won M., Herrera-Lavados C., Moreno-Osorio L. (2024). Influence of starch-protein interactions on the digestibility and chemical properties of a 3D-printed food matrix based on salmon by-product proteins. Food Research International.

[bb0050] Chakraborty I.N.P., Mal S.S., Paul U.C., Md Rahman H., Mazumder N. (2022). An insight into the gelatinization properties influencing the modified starches used in food industry: A review. Food and Bioprocess Technology.

[bb0055] Chang Q., Zheng B., Zhang Y., Zeng H. (2021). A comprehensive review of the factors influencing the formation of retrograded starch. International Journal of Biological Macromolecules.

[bb0060] Chatpapamon C., Wandee Y., Uttapap D., Puttanlek C., Rungsardthong V. (2019). Pasting properties of cassava starch modified by heat-moisture treatment under acidic and alkaline pH environments. Carbohydrate Polymers.

[bb0065] Chen P., Xie Q.-T., Wang R.-M., Wang S.-Y., Cheng J., Zhang B. (2022). Effects of pullulanase enzymatic hydrolysis on the textural of acorn vermicelli and its influencing mechanism on the quality. Food Research International.

[bb0070] Chen Y., McClements D.J., Peng X., Chen L., Xu Z., Meng M., Jin Z. (2024). Starch as edible ink in 3D printing for food applications: A review. Critical Reviews in Food Science and Nutrition.

[bb0075] Chen Y., Wang C., Chang T., Shi L., Yang H., Cui M. (2014). Effect of salts on textural, color, and rheological properties of potato starch gels. Starch - Stärke.

[bb0080] Chen Z., Hu A., Ihsan A., Zheng J. (2024). The formation, structure, and physicochemical characteristics of starch-lipid complexes and the impact of ultrasound on their properties: A review. Trends in Food Science & Technology.

[bb0085] Cheng F., Ren Y., Warkentin T.D., Ai Y. (2024). Heat-moisture treatment to modify structure and functionality and reduce digestibility of wrinkled and round pea starches. Carbohydrate Polymers.

[bb0090] Cheng H., Chen L., Zhang Z., Zhang R., McClements D.J., Xu H., Jin Z. (2023). Effect of addition of pullulan on the properties of native/waxy corn starch-based films. Food Bioengineering.

[bb0095] Cheng Y., Liang K., Chen Y., Gao W., Kang X., Li T., Cui B. (2023). Effect of molecular structure changes during starch gelatinization on its rheological and 3D printing properties. Food Hydrocolloids.

[bb0100] Chi C., Xu K., Wang H., Zhao L., Zhang Y., Chen B., Wang M. (2023). Deciphering multi-scale structures and pasting properties of wheat starch in frozen dough following different freezing rates. Food Chemistry.

[bb0105] Chi Y., Maitland E., Pascall M.A. (2024). The effect of citric acid concentrations on the mechanical, thermal, and structural properties of starch edible films. International Journal of Food Science & Technology.

[bb0110] Deng C., Melnyk O., Luo Y. (2022). Optimization of heat-moisture treatment on potato starch and study on its physicochemical properties. Technology Audit and Production Reserves.

[bb0115] Du J., Qi Y., Liu S., Xu B. (2024). Potential relation between starch granule-associated proteins and retrogradation properties of buckwheat starch. International Journal of Biological Macromolecules.

[bb0120] Falsafi S.R., Maghsoudlou Y., Rostamabadi H., Rostamabadi M.M., Hamedi H., Hosseini S.M.H. (2019). Preparation of physically modified oat starch with different sonication treatments. Food Hydrocolloids.

[bb0125] Fan Z., Cheng P., Zhang P., Zhang G., Han J. (2022). Rheological insight of polysaccharide/protein based hydrogels in recent food and biomedical fields: A review. International Journal of Biological Macromolecules.

[bb0130] Fang F., Luo X., Fei X., Mathews M.A.A., Lim J., Hamaker B.R., Campanella O.H. (2020). Stored gelatinized waxy potato starch forms a strong retrograded gel at low pH with the formation of intermolecular double helices. Journal of Agricultural and Food Chemistry.

[bb0135] Geng D.-H., Tang N., Gan J., Cheng Y. (2024). Two-step modification of pullulanase and transglucosidase: A novel way to improve the gel strength and reduce the digestibility of rice starch. International Journal of Biological Macromolecules.

[bb0140] Geng D.-H., Zhang X., Zhu C., Wang C., Cheng Y., Tang N. (2023). Structural, physicochemical and digestive properties of rice starch modified by preheating and pullulanase treatments. Carbohydrate Polymers.

[bb0145] Gong Y., Xu S., He T., Dong R., Ren T., Wang X., Hu X. (2020). Effect of quick-freezing temperature on starch retrogradation and ice crystals properties of steamed oat roll. Journal of Cereal Science.

[bb0150] Gu Z., Sha X., Wang X., Zhao R., Khashaba R., Jane J., Jiang H. (2024). Exploring characteristics of waxy wheat starches cross-linked at low levels: Insights from pasting, rheological, and textural properties. Food Bioscience.

[bb0155] Han X., Liang Q., Rashid A., Qayum A., Rehman A., Zhong M., Sun Y., Liu Y., Ma H., Miao S., Ren X. (2024). The effects of different hydrocolloids on lotus root starch gelatinization and gels properties. International Journal of Biological Macromolecules.

[bb0160] Hedayati S., Shahidi F., Koocheki A., Farahnaky A., Majzoobi M. (2016). Physical properties of pregelatinized and granular cold water swelling maize starches at different pH values. International Journal of Biological Macromolecules.

[bb0165] Hirao K., Kondo T., Kainuma K., Takahashi S. (2021). Starch gel foods in cookery science: Application of native starch and modified starches. Journal of Biorheology.

[bb0170] Hu A., Chen X., Wang J., Wang X., Zheng J., Wang L. (2021). Effects on the structure and properties of native corn starch modified by enzymatic debranching (ED), microwave assisted esterification with citric acid (MCAE) and by the dual ED/MCAE treatment. International Journal of Biological Macromolecules.

[bb0175] Hu H., Lin H., Xiao L., Guo M., Yan X., Su X., Sang S. (2022). Impact of native form oat β-glucan on the physical and starch digestive properties of whole oat bread. Foods.

[bb0180] Hu P., Fan X., Lin L., Wang J., Zhang L., Wei C. (2017). Effects of surface proteins and lipids on molecular structure, thermal properties, and enzymatic hydrolysis of rice starch. Food Science and Technology.

[bb0185] Hu W.-X., Chen J., Zhao J.-W., Chen L., Wang Y.-H. (2020). Effect of the addition of modified starch on gelatinization and gelation properties of rice flour. International Journal of Biological Macromolecules.

[bb0190] Huang J., Wu W., Niu B., Fang X., Chen H., Wang Y., Gao H. (2023). Characterization of Zizania latifolia polysaccharide-corn starch composite films and their application in the postharvest preservation of strawberries. LWT.

[bb0195] Huang S., Chao C., Yu J., Copeland L., Wang S. (2021). New insight into starch retrogradation: The effect of short-range molecular order in gelatinized starch. Food Hydrocolloids.

[bb0200] Ichihara T., Fukuda J., Takaha T., Suzuki S., Yuguchi Y., Kitamura S. (2016). Small-angle X-ray scattering measurements of gel produced from α-amylase-treated cassava starch granules. Food Hydrocolloids.

[bb0205] Ji X., Wang Z., Jin X., Qian Z., Qin L., Guo X., Yin M., Liu Y. (2022). Effect of inulin on the pasting and retrogradation characteristics of three different crystalline starches and their interaction mechanism. Frontiers in Nutrition.

[bb0210] Jia R., Cui C., Gao L., Qin Y., Ji N., Dai L., Wang Y., Xiong L., Shi R., Sun Q. (2023). A review of starch swelling behavior: Its mechanism, determination methods, influencing factors, and influence on food quality. Carbohydrate Polymers.

[bb0215] Jiang J., Zeng J., Gao H., Zhang L., Wang F., Su T., Xiang F., Li G. (2020). Effect of low temperature on the aging characteristics of a potato starch gel. International Journal of Biological Macromolecules.

[bb0220] Karma V., Gupta A.D., Yadav D.K., Singh A.A., Verma M., Singh H. (2022). Recent developments in starch modification by organic acids: A review. Starch - Stärke.

[bb0225] Kou T., Song J., Liu M., Fang G. (2022). Effect of amylose and crystallinity pattern on the gelatinization behavior of cross-linked starches. Polymers.

[bb0230] Kumar Y., Singh L., Sharanagat V.S., Patel A., Kumar K. (2020). Effect of microwave treatment (low power and varying time) on potato starch: Microstructure, thermo-functional, pasting and rheological properties. International Journal of Biological Macromolecules.

[bb0235] Lauer M.K., Smith R.C. (2020). Recent advances in starch-based films toward food packaging applications: Physicochemical, mechanical, and functional properties. Comprehensive Reviews in Food Science and Food Safety.

[bb0240] Lee H., Yoo B. (2023). Particle agglomeration and properties of Pregelatinized potato starch powder. Gels.

[bb0245] Li C. (2022). Recent progress in understanding starch gelatinization—An important property determining food quality. Carbohydrate Polymers.

[bb0250] Li C., Hu Y., Huang T., Gong B., Yu W.-W. (2020). A combined action of amylose and amylopectin fine molecular structures in determining the starch pasting and retrogradation property. International Journal of Biological Macromolecules.

[bb0255] Li C., Hu Y., Li E. (2021). Effects of amylose and amylopectin chain-length distribution on the kinetics of long-term rice starch retrogradation. Food Hydrocolloids.

[bb0260] Li C., Yu W., Gilbert R.G. (2022). The effects of starch molecular fine structure on thermal and digestion properties of Rice starch. Foods.

[bb0265] Li E., Lv J., Huo D., Jia B., Li C. (2023). Importance of amylose chain-length distribution in determining starch gelatinization and retrogradation property of wheat flour in the presence of different salts. Carbohydrate Polymers.

[bb0270] Li E., Yang X., Li C. (2022). Combined effects of starch fine molecular structures and storage temperatures on long-term rice amylopectin retrogradation property. International Journal of Biological Macromolecules.

[bb0275] Li G., Ge X., Guo C., Liu B. (2023). Effect of ultrasonic treatment on structure and physicochemical properties of pea starch. Foods.

[bb0280] Li H., Hu K., Liu X., Wang W., Chen J., Hu Y. (2023). Effects of *Lycium barbarum* polysaccharide on gelatinization properties of potato starch. Journal of Food Process Engineering.

[bb0285] Li H., Jiang Y., Yang J., Pang R., Chen Y., Mo L., Jiang Q., Qin Z. (2023). Preparation of curcumin-chitosan composite film with high antioxidant and antibacterial capacity: Improving the solubility of curcumin by encapsulation of biopolymers. Food Hydrocolloids.

[bb0290] Li H., Lei N., Yan S., Yang J., Yu T., Wen Y., Wang J., Sun B. (2019). The importance of amylopectin molecular size in determining the viscoelasticity of rice starch gels. Carbohydrate Polymers.

[bb0295] Li Q., Liu S., Obadi M., Jiang Y., Zhao F., Jiang S., Xu B. (2020). The impact of starch degradation induced by pre-gelatinization treatment on the quality of noodles. Food Chemistry.

[bb0300] Li Q., Zhang L., Ye Y., Gao Q. (2015). Effect of salts on the gelatinization process of Chinese yam (Dioscorea opposita) starch with digital image analysis method. Food Hydrocolloids.

[bb0305] Li X., Zhang X., Lv J., Zhang X., Li Y., Han X., Zhang W. (2024). Development of starch-based films reinforced with curcumin-loaded nanocomplexes: Characterization and application in the preservation of blueberries. International Journal of Biological Macromolecules.

[bb0310] Li X.-M., Wu Z.-Z., Zhang B., Pan Y., Meng R., Chen H.-Q. (2019). Fabrication of chitosan hydrochloride and carboxymethyl starch complex nanogels as potential delivery vehicles for curcumin. Food Chemistry.

[bb0315] Li Y., Cheng W., Qiu X., Sun Y., Xia X., Yang L., Fan M., Wang L., Qian H. (2023). Effects of β-amylase hydrolysis on the structural, physicochemical and storage properties of wheat starch. Journal of Cereal Science.

[bb0320] Li Y., Hu A., Wang X., Zheng J. (2019). Physicochemical and in vitro digestion of millet starch: Effect of moisture content in microwave. International Journal of Biological Macromolecules.

[bb0325] Liang B., Yin X., Zhang W., Shang S., Zeng S., Wei H. (2024). A study on influences of an induced electric field on the Enzymolysis of water caltrop starch by Pullulanase. International Journal of Food Science & Technology.

[bb0330] Liu D., Zhao P., Chen J., Yan Y., Wu Z. (2022). Recent advances and applications in starch for intelligent active food packaging: A review. Foods.

[bb0335] Liu S., Yang T., Ming T.W., Gaun T.K.W., Zhou T., Wang S., Ye B. (2020). Isosteroid alkaloids with different chemical structures from Fritillariae cirrhosae bulbus alleviate LPS-induced inflammatory response in RAW 264.7 cells by MAPK signaling pathway. International Immunopharmacology.

[bb0340] Liu W., Pan W., Li J., Chen Y., Yu Q., Rong L., Xiao W., Wen H., Xie J. (2022). Dry heat treatment induced the gelatinization, rheology and gel properties changes of chestnut starch. Current Research in Food Science.

[bb0345] Liu Y., Chen X., Xu Y., Xu Z., Li H., Sui Z., Corke H. (2021). Structural characterization and immunomodulatory activity of a new polysaccharide isolated from the radix of Platycodon grandiflorum. International Journal of Food Science & Technology.

[bb0350] Liu Z., Zhong Y., Khakimov B., Fu Y., Pawel Czaja T., Judas Kain Kirkensgaard J., Blennow A., Shen Q., Balling Engelsen S. (2023). Insights into high hydrostatic pressure pre-treatment generating a more efficient catalytic mode of maltogenic α-amylase: Effect of multi-level structure on retrogradation properties of maize starch. Food Hydrocolloids.

[bb0355] Lowithun N., Sagis L.M.C., Lumdubwong N. (2024). Impact of deformability and rigidity of starch granules on linear and non-linear rheological behavior of waxy Rice starch gels and applicability for food end uses. Foods.

[bb0360] Luo Z., Wang Z. (2022). The role of starch granule-associated proteins in enhancing the strength of indica rice starch gels. Food Hydrocolloids.

[bb0365] Ma H., Liu M., Liang Y., Zheng X., Sun L., Dang W., Li J., Li L., Liu C. (2022). Research progress on properties of pre-gelatinized starch and its application in wheat flour products. Grain & Oil Science and Technology.

[bb0370] Ma M., Zhu H., Liu Z., Sui Z., Corke H. (2022). Removal of starch granule-associated proteins alters the physicochemical properties of diverse small granule starches. Food Hydrocolloids.

[bb0375] Maniglia B.C., Lima D.C., Da Matta Júnior M., Oge A., Le-Bail P., Augusto P.E.D., Le-Bail A. (2020). Dry heating treatment: A potential tool to improve the wheat starch properties for 3D food printing application. Food Research International.

[bb0380] Martinez M.M., Li C., Okoniewska M., Mukherjee I., Vellucci D., Hamaker B. (2018). Slowly digestible starch in fully gelatinized material is structurally driven by molecular size and A and B1 chain lengths. Carbohydrate Polymers.

[bb0385] Mendonça A.P.S., Dos Reis K.L., Barbosa-Tessmann I.P. (2023). Aspergillus clavatus UEM 04: An efficient producer of glucoamylase and α-amylase able to hydrolyze gelatinized and raw starch. International Journal of Biological Macromolecules.

[bb0390] Meng R., Wu Z., Xie H.-Q., Xu G.-X., Cheng J.-S., Zhang B. (2020). Preparation, characterization, and encapsulation capability of the hydrogel cross-linked by esterified tapioca starch. International Journal of Biological Macromolecules.

[bb0395] Mohamed I.O. (2023). Interaction of starch with some food macromolecules during the extrusion process and its effect on modulating physicochemical and digestible properties. A review. Carbohydrate Polymer Technologies and Applications.

[bb0400] Nagasaki A., Matsuba G., Ikemoto Y., Moriwaki T., Ohta N., Osaka K. (2021). Analysis of the sol and gel structures of potato starch over a wide spatial scale. Food Science & Nutrition.

[bb0405] Navaf M., Sunooj K.V., Krishna N.U., Aaliya B., Sudheesh C., Akhila P.P., George J. (2022). Effect of different hydrothermal treatments on pasting, textural, and rheological properties of single and dual modified *Corypha Umbraculifera* L. Starch. Starch - Stärke.

[bb0410] Negi A., Barthwal R., Kathuria D., Singh N. (2024). Enzymatic advances in starch modification: Creating functional derivatives and exploring applications. Food Bioscience.

[bb0415] Obadi M., Chen Y., Qi Y., Liu S., Xu B. (2020). Effects of different pre-gelatinized starch on the processing quality of high value-added Tartary buckwheat noodles. Journal of Food Measurement and Characterization.

[bb0420] Obadi M., Xu B. (2021). Review on the physicochemical properties, modifications, and applications of starches and its common modified forms used in noodle products. Food Hydrocolloids.

[bb0425] Oh I.K., Bae I.Y., Lee H.G. (2018). Effect of dry heat treatment on physical property and in vitro starch digestibility of high amylose rice starch. International Journal of Biological Macromolecules.

[bb0430] Ouyang J., Wang C., Huang Q., Guan Y., Zhu Z., He Y., Jiang G., Xiong Y., Li X. (2024). Correlation between in vitro starch digestibility and starch structure/physicochemical properties in rice. International Journal of Biological Macromolecules.

[bb0435] Oyeyinka S.A., Akintayo O.A., Adebo O.A., Kayitesi E., Njobeh P.B. (2021). A review on the physicochemical properties of starches modified by microwave alone and in combination with other methods. International Journal of Biological Macromolecules.

[bb0440] Pan W., Qi X., Shen M., Chen Y., Yu Q., Huang Z., Xie J. (2024). Effects of synergistic modification using alkalis and guar gum on the pasting, rheological, and microstructural properties of germinated highland barley starch gels. Food Chemistry.

[bb0445] Peng Q., Ma Y., Wang Z., Wang J. (2024). Inhibition mechanism of different structural polyphenols against α-amylase studied by solid-state NMR and molecular docking. International Journal of Biological Macromolecules.

[bb0450] Punia Bangar S., Ashogbon A.O., Singh A., Chaudhary V., Whiteside W.S. (2022). Enzymatic modification of starch: A green approach for starch applications. Carbohydrate Polymers.

[bb0455] Punia Bangar S., Sunooj K.V., Navaf M., Phimolsiripol Y., Whiteside W.S. (2024). Recent advancements in cross-linked starches for food applications- a review. International Journal of Food Properties.

[bb0460] Qiao J., Jia M., Niu J., Zhang Z., Xing B., Liang Y., Li H., Zhang Y., Ren G., Qin P., Zhang L. (2024). Amylopectin chain length distributions and amylose content are determinants of viscoelasticity and digestibility differences in mung bean starch and proso millet starch. International Journal of Biological Macromolecules.

[bb0465] Qiao K., Peng B. (2024). Freezing rate’s impact on starch retrogradation, ice recrystallization, and quality of water-added and water-free quick-frozen rice noodles. International Journal of Biological Macromolecules.

[bb0470] Qiu C., Cao J., Xiong L., Sun Q. (2015). Differences in physicochemical, morphological, and structural properties between rice starch and rice flour modified by dry heat treatment. Starch - Stärke.

[bb0475] Radi M., Abedi E., Najafi A., Amiri S. (2022). The effect of freezing-assisted cross-linking on structural and rheological properties of potato starch. International Journal of Biological Macromolecules.

[bb0480] Ren Y., Rong L., Shen M., Liu W., Xiao W., Luo Y., Xie J. (2020). Interaction between rice starch and Mesona chinensis Benth polysaccharide gels: Pasting and gelling properties. Carbohydrate Polymers.

[bb0485] Reyniers S., Vluymans N., De Brier N., Ooms N., Matthijs S., Brijs K., Delcour J.A. (2020). Amylolysis as a tool to control amylose chain length and to tailor gel formation during potato-based crisp making. Food Hydrocolloids.

[bb0490] Ribotta P.D., Colombo A., Rosell C.M. (2012). Enzymatic modifications of pea protein and its application in protein–cassava and corn starch gels. Food Hydrocolloids.

[bb0495] Rong L., Chen X., Shen M., Yang J., Qi X., Li Y., Xie J. (2023). The application of 3D printing technology on starch-based product: A review. Trends in Food Science & Technology.

[bb0500] Rostamabadi H., Bajer D., Demirkesen I., Kumar Y., Su C., Wang Y., Falsafi S.R. (2023). Starch modification through its combination with other molecules: Gums, mucilages, polyphenols and salts. Carbohydrate Polymers.

[bb0505] Schafranski K., Ito V.C., Lacerda L.G. (2021). Impacts and potential applications: A review of the modification of starches by heat-moisture treatment (HMT). Food Hydrocolloids.

[bb0510] Scott G., Awika J.M. (2023). Effect of protein–starch interactions on starch retrogradation and implications for food product quality. Comprehensive Reviews in Food Science and Food Safety.

[bb0515] Shah A., Wang Y., Tao H., Zhang W., Cao S. (2023). Insights into the structural characteristics and in vitro starch digestibility on parboiled rice as affected by ultrasound treatment in soaking process. Food Chemistry: X.

[bb0525] Sharma V., Kaur M., Sandhu K.S., Kaur S., Nehra M. (2021). Barnyard millet starch cross-linked at varying levels by sodium trimetaphosphate (STMP): Film forming, physico-chemical, pasting and thermal properties. Carbohydrate Polymer Technologies and Applications.

[bb0530] Shen H., Yu J., Bai J., Liu Y., Ge X., Li W., Zheng J. (2023). A new pre-gelatinized starch preparing by spray drying and electron beam irradiation of oat starch. Food Chemistry.

[bb0535] Sindhu R., Devi A., Khatkar B.S. (2019). Physicochemical, thermal and structural properties of heat moisture treated common buckwheat starches. Journal of Food Science and Technology.

[bb0540] Siwatch M., Yadav R.B., Yadav B.S. (2022). Annealing and heat-moisture treatment of amaranth starch: Effect on structural, pasting, and rheological properties. Journal of Food Measurement and Characterization.

[bb0545] Song J., Rong L., Li J., Shen M., Yu Q., Chen Y., Kong J., Xie J. (2024). Effects of three different polysaccharides on the sol gel-behavior, rheological, and structural properties of tapioca starch. International Journal of Biological Macromolecules.

[bb0550] Sun B., Qian X., Zhou M., Gu Y., Ma S., Wang X. (2023). Changes of gelation behavior, water distribution and digestibility of protein-starch mixtures in the oat dough/batter model affected by water. LWT.

[bb0555] Tang J., Zou F., Guo L., Wang N., Zhang H., Cui B., Liu X. (2022). The relationship between linear chain length distributions of amylopectin and the functional properties of the debranched starch-based films. Carbohydrate Polymers.

[bb0560] Tao H., Huang L.-J., Li S., Lu F., Cai W.-H., Wang H.-L. (2024). Insight into the promoted recrystallization and water distribution of bread by removing starch granule—Surface and—Associated proteins during storage. Food Chemistry.

[bb0565] Tao Y., Yan B., Fan D., Zhang N., Ma S., Wang L., Wu Y., Wang M., Zhao J., Zhang H. (2020). Structural changes of starch subjected to microwave heating: A review from the perspective of dielectric properties. Trends in Food Science & Technology.

[bb0570] Tian J., Qin L., Zeng X., Ge P., Fan J., Zhu Y. (2023). The role of amylose in gel forming of Rice flour. Foods.

[bb0575] Torres M.D., Chenlo F., Moreira R. (2018). Rheological effect of gelatinisation using different temperature-time conditions on potato starch dispersions: Mechanical characterisation of the obtained gels. Food and Bioprocess Technology.

[bb0580] Ulbrich M., Scholz F., Braun B., Bussert R., Flöter E. (2023). High amylose corn starch gels—Investigation of the Supermolecular structure. Starch - Stärke.

[bb0585] Wang B., Yu B., Yuan C., Guo L., Liu P., Gao W., Abd El-Aty A.M. (2022). An overview on plasticized biodegradable corn starch-based films: The physicochemical properties and gelatinization process. Critical Reviews in Food Science and Nutrition.

[bb0590] Wang H., Xiao N., Wang X., Zhao X., Zhang H. (2019). Effect of pregelatinized starch on the characteristics, microstructures, and quality attributes of glutinous rice flour and dumplings. Food Chemistry.

[bb0595] Wang L., Zhu L., Gao J., Zhang F., Li L., Yang Y., Xu Y. (2022). Effect of dandelion root polysaccharide on structure, rheology and retrogradation properties of corn starch during storage. International Journal of Food Science & Technology.

[bb0600] Wang N., You Y., Liao X., Zhang F., Kan J., Zheng J. (2023). Ultrasonic modification of lotus starch based on multi-scale structure: Pasting, rheological, and thermal properties. LWT.

[bb0605] Wang Q., Li L., Zheng X. (2021). Recent advances in heat-moisture modified cereal starch: Structure, functionality and its applications in starchy food systems. Food Chemistry.

[bb0610] Wang S., Li C., Copeland L., Niu Q., Wang S. (2015). Starch Retrogradation: A comprehensive review. Comprehensive Reviews in Food Science and Food Safety.

[bb0615] Wang S., Zhang X., Wang S., Copeland L. (2016). Changes of multi-scale structure during mimicked DSC heating reveal the nature of starch gelatinization. Scientific Reports.

[bb0620] Wang T., Qin Y., Cui C., Ji N., Dai L., Wang Y., Xiong L., Shi R., Sun Q. (2023). The effects of pH and iron ions on the mechanical properties of pea starch hydrogels. International Journal of Biological Macromolecules.

[bb0625] Wang X., Liu S., Ai Y. (2022). Gelation mechanisms of granular and non-granular starches with variations in molecular structures. Food Hydrocolloids.

[bb0630] Wang Y., Bai Y., Ji H., Dong J., Li X., Liu J., Jin Z. (2022). Insights into rice starch degradation by maltogenic α–amylase: Effect of starch structure on its rheological properties. Food Hydrocolloids.

[bb0635] Wang Y., Liu Q., Yang Y., Qiu C., Jiao A., Jin Z. (2023). Impact of pH on pea protein–hydroxypropyl starch hydrogel based on interpenetrating network and its application in 3D-printing. Food Research International.

[bb0640] Wedamulla N.E., Fan M., Choi Y.-J., Kim E.-K. (2023). Combined effect of heating temperature and content of pectin on the textural properties, rheology, and 3D printability of potato starch gel. International Journal of Biological Macromolecules.

[bb0645] Wei W., Wu M., Ren W., Yu H., Sun D. (2024). Preparation of crosslinked starches with enhanced and tunable gel properties by the cooperative crosslinking-extrusion combined modification. Carbohydrate Polymers.

[bb0650] Wu C., Gong X., Zhang J., Zhang C., Qian J.-Y., Zhu W. (2023). Effect of rice protein on the gelatinization and retrogradation properties of rice starch. International Journal of Biological Macromolecules.

[bb0655] Xiao W., Shen M., Ren Y., Rong L., Liu W., Chen X., Yang J., Li J., Xie J. (2021). Mesona chinensis polysaccharides promote molecular crosslinking and gel formation of debranched waxy maize starch. LWT.

[bb0660] Xie J., Wei S., Xu X., Xu D., Jin Y., Yang N., Wu F. (2022). Preparation, structure, and properties of enzymatically-hydrolyzed starch for slowing down the Retrogradation of high starchy foods. Starch - Stärke.

[bb0665] Xie Y., Yan M., Yuan S., Sun S., Huo Q. (2013). Effect of microwave treatment on the physicochemical properties of potato starch granules. Chemistry Central Journal.

[bb0670] Xu J., Blennow A., Li X., Chen L., Liu X. (2020). Gelatinization dynamics of starch in dependence of its lamellar structure, crystalline polymorphs and amylose content. Carbohydrate Polymers.

[bb0675] Xu K., Zhang M., Bhandari B. (2020). Effect of novel ultrasonic- microwave combined pretreatment on the quality of 3D printed wheat starch-papaya system. Food Biophysics.

[bb0680] Xu L., Wang T., Shan Y., Wang R., Yi C. (2024). Soybean protein isolate inhibiting the retrogradation of fresh rice noodles: Combined experimental analysis and molecular dynamics simulation. Food Hydrocolloids.

[bb0685] Xu L., Zhu H., Yi C. (2023). Soybean protein isolate affects in vitro digestion properties of fermented indica rice starch by regulating its gel characteristics. Food Hydrocolloids.

[bb0690] Xu M., Ji S., Li Y., Li J., Liu Y., Li K., Lu B. (2023). Exploring the mechanism of variation in 3D printing accuracy of cassava starch gels during freezing process. Food Hydrocolloids.

[bb0695] Xu Z., Liu X., Ma M., He J., Sui Z., Corke H. (2024). Reduction of starch granule surface lipids alters the physicochemical properties of crosslinked maize starch. International Journal of Biological Macromolecules.

[bb0700] Yang F., Du Q., Miao T., Zhang X., Xu W., Jia D. (2022). Interaction between potato starch and Tremella fuciformis polysaccharide. Food Hydrocolloids.

[bb0705] Yang X., Pan Y., Li S., Li C., Li E. (2022). Effects of amylose and amylopectin molecular structures on rheological, thermal and textural properties of soft cake batters. Food Hydrocolloids.

[bb0710] Yang Z., Wang Z., Liu P., Liu W., Xu Y., Zhou Y., Yu Z., Zheng M., Xiao Y., Liu Y. (2024). Development of dual-channel starch-based film incorporated with betanin@β-cyclodextrin inclusion complex and berberine for indicating shrimp freshness. Food Chemistry.

[bb0715] Yang Z., Zhang Y., Wu Y., Ouyang J. (2023). Factors influencing the starch digestibility of starchy foods: A review. Food Chemistry.

[bb0720] Ye S.-J., Baik M.-Y. (2023). Characteristics of physically modified starches. Food Science and Biotechnology.

[bb0725] Yu H., Chi S., Li D., Wang L., Wang Y. (2022). Effect of gums on the multi-scale characteristics and 3D printing performance of potato starch gel. Innovative Food Science & Emerging Technologies.

[bb0730] Yu S., Ma Y., Sun D.-W. (2010). Effects of freezing rates on starch retrogradation and textural properties of cooked rice during storage. LWT - Food Science and Technology.

[bb0735] Yu X., Wang L., Zhang J., Wang Z., Wang K., Duan Y., Xiao Z., Wang P. (2023). Understanding effects of glutelin on physicochemical and structural properties of extruded starch and the underlying mechanism. Carbohydrate Polymers.

[bb0740] Yu X., Wang P., Wang L., Wang K., Duan Y., Huo J., Ma X., Dong S., Xin G., Xiao Z. (2024). Inhibition mechanism of rice glutelin on extruded starch digestion: From the structural properties of starch and enzyme activity. Food Research International.

[bb0745] Yuan M., Wang Y., Bai Y., Birte S. (2022). Distinct effects of different α-amylases on cross-linked tapioca starch and gel-improving mechanism. Food Hydrocolloids.

[bb0750] Zhan Q., Ye X., Zhang Y., Kong X., Bao J., Corke H., Sui Z. (2020). Starch granule-associated proteins affect the physicochemical properties of rice starch. Food Hydrocolloids.

[bb0755] Zhang B., Xiao Y., Wu X., Luo F., Lin Q., Ding Y. (2021). Changes in structural, digestive, and rheological properties of corn, potato, and pea starches as influenced by different ultrasonic treatments. International Journal of Biological Macromolecules.

[bb0760] Zhang H., Wang R., Chen Z., Zhong Q. (2019). Enzymatically modified starch with low digestibility produced from amylopectin by sequential amylosucrase and pullulanase treatments. Food Hydrocolloids.

[bb0765] Zhang J., Kong H., Ban X., Li C., Gu Z., Li Z. (2022). Rice noodle quality is structurally driven by the synergistic effect between amylose chain length and amylopectin unit-chain ratio. Carbohydrate Polymers.

[bb0770] Zhang P., Wang Y., Liu Y., Wu Y., Ouyang J. (2023). Improved stability of β-carotene by encapsulation in SHMP-corn starch aerogels. Food Chemistry.

[bb0775] Zhang S., Zhu S., Zhong F., Huang D., Chen X., Li Y. (2023). Study on the mechanism of various exogenous proteins with different inhibitions on wheat starch digestion: From the distribution behaviors of protein in the starch matrix. International Journal of Biological Macromolecules.

[bb0780] Zhang Y., Chen C., Chen Y., Chen Y. (2019). Effect of rice protein on the water mobility, water migration and microstructure of rice starch during retrogradation. Food Hydrocolloids.

[bb0785] Zhao T., Zhang H., Chen F., Tong P., Cao W., Jiang Y. (2022). Study on structural changes of starches with different amylose content during gelatinization process. Starch - Stärke.

[bb0790] Zheng L., Li D., Wang L., Wang Y. (2024). Tailoring 3D-printed high internal phase emulsion-rice starch gels: Role of amylose in rheology and bioactive stability. Carbohydrate Polymers.

[bb0795] Zheng L., Ren A., Liu R., Xing Y., Yu X., Jiang H. (2022). Effect of sodium chloride solution on quality of 3D-printed samples molded using wheat starch gel. Food Hydrocolloids.

[bb0800] Zheng L., Zhang Q., Yu X., Luo X., Jiang H. (2023). Effect of annealing and heat-moisture pretreatment on the quality of 3D-printed wheat starch gels. Innovative Food Science & Emerging Technologies.

[bb0805] Zhong Y., Tian Y., Liu X., Ding L., Kirkensgaard J.J.K., Hebelstrup K., Blennow A. (2021). Influence of microwave treatment on the structure and functionality of pure amylose and amylopectin systems. Food Hydrocolloids.

[bb0810] Zhou X., Campanella O.H., Hamaker B.R., Miao M. (2021). Deciphering molecular interaction and digestibility in retrogradation of amylopectin gel networks. Food & Function.

